# Getting off the ladder: Disentangling water quality indices to enhance the valuation of divergent ecosystem services^*^

**DOI:** 10.1073/pnas.2120261120

**Published:** 2023-04-24

**Authors:** Frank Lupi, Joseph A. Herriges, Hyunjung Kim, R. Jan Stevenson

**Affiliations:** ^a^Department of Agricultural, Food, and Resource Economics, Michigan State University, East Lansing, MI 48824; ^b^Department of Fisheries and Wildlife, Michigan State University, East Lansing, MI 48824; ^c^Department of Economics, Michigan State University, East Lansing, MI 48824; ^d^Department of Integrative Biology, Michigan State University, East Lansing, MI 48824

**Keywords:** water quality indices, stated preferences, willingness to pay

## Abstract

Many water quality valuation studies and Federal cost–benefit analyses build from pioneering work using a “water quality ladder” or a single water quality index (WQI) to characterize both current conditions and effects of policies. When policies lead to contrasting changes in valued ecosystem services like recreational fishing and swimming, analyses using a single ladder or index might obscure important underlying service trade-offs. We test for this effect using alternative approaches that separate water quality indices and value changes in distinct ecosystem services stemming from policies with small to moderate changes in water quality. The indices we test relate to nutrient loadings in Michigan’s rivers, lakes, and Great Lakes. Our split-sample experiment compares economic values for treatments with two versus three quality metrics. The key distinction is that the two-index survey, like many existing studies, aggregates subindices for water contact (for swimming and boating) and fish biomass scores (for fishing) into a single WQI, whereas the three-index survey separately utilizes both. We find that changes in our index reflecting changes in fecal bacteria and water clarity are valued differently from changes in our recreational fishing index. Aggregating changes in these two distinct recreational services using a single WQI yields consistently lower benefit estimates across a range of underlying changes in our experiment. In valuation scenarios with small changes in overall water quality, the WQI-based benefit estimates can differ substantially from benefits measured by decomposing the index and valuing the disparate subindices, differences which might change balance of benefits and costs in regulatory evaluations.

## Significance

Benefit–cost analyses and ecosystem service assessments provide critical information that can guide society toward wiser stewardship of resources. When actions change multiple ecosystem services, representing the changes via single summaries or indices can mask underlying trade-offs between services. For some water quality scenarios, we find that combining ecosystem service metrics together into one index can yield lower benefit estimates, potentially changing relative costs and benefits. Consequently, even for very small changes in water quality, benefit estimates can differ substantially from benefits measured by decomposing the index and separately valuing the disparate ecosystem services.

Managing and protecting ecosystems and their services often involves trade-offs between ecosystem services (1–3). Economics offers benefit–cost analysis (BCA) as a framework to assess trade-offs by comparing economic benefits to costs to understand the net economic value to society, including when these benefits and costs include changes in ecosystem service values and other nonmarket effects (1, 4, 5). Similarly, Federal agencies in the United States use BCA to evaluate regulations that may have a significant economic impact (6). In cases involving water resources and services, even when a decision would be beneficial to society, it can have negative effects on a particular service such as water quality (1). This paper tests the effect of underlying ecosystem trade-offs that can exist when conducting or applying economic valuation of water quality changes.

For water quality valuation, many studies build off pioneering work of Carson and Mitchell (7), using a “water quality ladder” and/or a single water quality index (WQI) (8–10) to characterize both current conditions and potential policy scenarios. This literature forms the backbone of several important meta-analyses and national benefit–costs evaluations of water quality regulations (6, 10). The obvious advantage of the water quality ladder is the simplicity of the metric, with its “fishable,” “boatable,” and “swimmable” rungs, making it relatively easy to convey to survey respondents. However, actual policies often result in fairly small changes in water quality that do not easily map into ladder steps, yet large water quality changes undergird meta-analyses of values.

Although the ladder or WQI can sometimes be tied to underlying physical measures of water quality such as nutrient concentrations (9), they can mask critical biological realities that are key to disentangling trade-offs among ecosystem services (6). For example, some recreationally targeted game fish are abundant and thrive in waters with high levels of nutrients, yet such waters can be poorly suited for swimming. In freshwater systems, nutrients are stressors, and at low levels, increases can benefit gamefish production, even though at high levels, nutrient increases can harm some gamefish species (11, 12). Since gamefish production is a valued ecosystem service, this particular stressor (nutrients) can cause trade-offs between valued services like fishing and water clarity. Yet, a single ladder or an index that combines these sometimes-conflicting services will mask potentially important trade-offs among these services. In addition, the fact that much of the water quality valuation literature has incomplete connections to ecological production functions and often omits passive-use values connected to small changes in water quality may partially explain the low benefit–cost ratios found in many cost–benefit analyses (13).

This paper examines an alternative approach designed to separate and value some of the potentially disparate changes in aquatic ecosystem services stemming from a policy scenario. Specifically, we estimate total values for small to moderate changes in water quality in Michigan’s rivers, inland lakes, and Great Lakes with an interest in services affected by nutrient loading. We utilize multiple water quality metrics reflecting key distinct aquatic ecosystem services, which in turn are linked to underlying nutrient loads. These water quality indices break out services into 1) a modified WQI that focuses on non-fishing water-based recreation (our “water contact score,” WCS), 2) a gamefish biomass index (our “fish biomass score”, FBS), and 3) an index of the biological condition of all aquatic wildlife (our wildlife score, WLS).

We then conduct a split-sample contingent valuation exercise to compare values elicited using two survey treatments that use two versus three indices to describe current and proposed water quality conditions. Both treatments separate the WLS into a distinct index, but the key distinction in treatments is that the two-index survey, like much of the existing literature, aggregates the recreational services captured by water contact activities (for swimming and boating) and fish biomass (for recreational fishing) into a single WQI, whereas the three-index survey reports them separately. In both treatments, the metrics are derived from the same underlying biophysical data, ecological models, and judgments. Similarly, both treatments use the same experimental design, which enables a wide range of potential tests for consistency, including nonlinear nested tests. Our results reveal that changes in water contact attributes (captured by our WCS through changes in bacteria and clarity) are valued differently from changes in fish biomass. We find that aggregating changes to these two distinct ecosystem services using a single WQI can mask these differences and lead to substantial and statistically significant differences for valuing some policy scenarios. The results are consistent with simulations of a WQI that were done for actual U.S. Environmental Protection Agency (EPA) benefit–cost analyses of Federal regulations that suggested that aggregations of subindices into a WQI can have large effects on the benefit estimation, potentially changing the balance of benefits and costs (14).

## Development and Use of Water Quality Indices

Water quality indices, and the water quality ladder, have a long history as tools for succinctly characterizing conditions in the nation’s water bodies (6, 15, 16). The EPA’s WQI, in particular, builds off of early work by McClelland (8). Therein, the author used a series of expert opinion surveys to link measurable water quality parameters (e.g., fecal coliform, total nitrogen, and dissolved oxygen) to an index of overall water quality.

The resulting water quality indices developed over more than a half-century have much to recommend them for use in describing water quality conditions and the impact of proposed policy changes. Particularly when coupled with the water quality ladder, they provide a simple summary of conditions that both policymakers and the public (e.g., including survey respondents) can readily relate to, with their swimmable, fishable, and boatable designated use categories. Moreover, they are linked to observable water quality measures, incorporating expert opinions regarding how these water quality metrics contribute to overall water quality. The WQI has been widely applied in economic valuation as well as meta-analyses. For example, 51 unique WQI studies were used in meta-analyses of models of Johnston et al. (17, 18). The WQI is also widely used in past EPA assessments of cost and benefits of regulations (6), and EPA researchers have developed a WQI-based integrated assessment model for future uses (19).

These advantages, however, come at a cost. First, the indices can portray water quality changes as a unidimensional concept and that impact of potentially conflicting changes in the underlying components of the WQI could be masked by the single-valued result. This could fail to capture key differences in the underlying ecosystem services people care about. In particular, in some regions or for some policies, significant reductions in nutrients may enhance the use of water bodies for valuable services such as swimming or boating, while at the same time, reducing the ability to support the same levels of key recreational gamefish.

For example, changes in phosphorus concentrations, across the range of concentrations in most Michigan streams and lakes, would have opposite effects on WCS and WLS compared to FBS. With low phosphorus limiting productivity of most Michigan streams and lakes, an increase in phosphorus would increase algal growth, reduce water clarity, increase the risk of cyanobacterial blooms, and decrease biological condition (20–24). Thus, increases in phosphorus cause lower WCS and WLS. In contrast, increasing phosphorus in waterbodies with relatively low phosphorus concentrations, which is the case in most Michigan streams and lakes, could increase gamefish fish growth and reproduction for most species (FBS) due to the greater algal productivity and biomass of the invertebrates eating algae (11, 25–29). Fish species may change, causing a decrease in biological condition of all fish with increasing phosphorus (12, 30), even though the biomass of most game fish will generally increase in Michigan streams and lakes that have low, production-limiting phosphorus concentrations (See *SI Appendix*, section 1.5 for an in-depth discussion of all these ecological relationships).

Second, while the WQI is linked to underlying water quality parameters, such as phosphorus and nitrates, the surveys used to elicit these connections are defined in terms of a vague notion of “overall water quality,” not the more specific ecosystem services (31) that consumers ultimately value. Moreover, the weights (*w_i_* in WQI function) given to component water quality parameters are based on largely ad hoc aggregations of the five-point importance ratings that individual experts assigned to each parameter (*SI Appendix*, section 1.0).

Third, the traditional WQI emphasizes the “use” values associated with changing ecosystem services (swimming, boating, etc.), potentially ignoring the underlying ecological conditions that may contribute to the nonuse values of a water body. Yet, these nonuse values may represent a substantial portion of the overall benefits stemming from a proposed policy scenario. Several authors have sought to remedy this limitation in recent years. One approach, advocated by Hime et al. (32), is to redefine the rungs of the water quality ladder so that they also capture the ecological health of a water body. Specifically, they replace the boatable, fishable, swimmable, and drinkable water quality levels in the traditional ladder with four color-coded levels of water quality (increasing from red to yellow to green to blue). These levels are defined not only in terms of fish stock and water clarity but also in terms of aquatic vegetation, bank vegetation, and substrate composition. The advantage of this approach is that the new water quality ladder reflects ecological conditions at a site, with policies defined in terms of color-coded maps depicting the distribution of water bodies falling into the four water quality levels. A disadvantage of this approach is that it allows for only four ecological endpoints in describing water quality. Just as was the case with the traditional water quality ladder, the expanded ladder implicitly masks underlying, potentially relevant, trade-offs in ecosystem services.

A second approach, and the one pursued here, is to employ multiple water quality indices to reflect different aspects of water quality. This is the approach being used by the EPA to develop a National Water Quality valuation approach (33). Specifically, they distinguish the impact of a policy scenario on recreational ecosystem services characterized via a “Recreation Score” from its impact on biological conditions characterized via a “WLS.” The Recreation Score is essentially the traditional WQI, emphasizing use-based ecosystem services, while the WLS measures the impact of policies on the underlying ecological conditions, measuring the ability of a waterbody to support healthy and diverse populations of aquatic plants and animals akin to healthy waterbodies indices and biological condition gradients (34). In this paper, we expand on a two-index approach by distinguishing two WQI subindices to reflect two types of use-based recreation: a Fishing Biomass Score (FBS) to capture fishing conditions and a WCS for nonfishing recreation such as swimming and boating. This allows for the possibility that changes in underlying water quality parameters can differentially impact these two types of recreation and, more importantly, that consumers might care about the differences. In the next section, we describe in more detail the construction of the individual water quality indices and the contingent valuation experiment that we designed to evaluate their use.

## Contingent Valuation Survey Experiment

For our experiment, we use an approach to economic valuation of changes in the environment based on contingent valuation, which is a survey-based method to elicit people’s preferences. The survey describes a possible change in water quality (due to what we refer to as a “plan”) that comes at a cost to the household. Survey respondents vote for or against the plan they are shown, and because the changes in cost and water quality are varied across the sample, the method allows statistical estimation of the effect each parameter has on each household’s willingness to forgo income for a water quality change, a trade-off that reveals the willingness to pay (WTP) for the water quality change.

Since our contingent valuation study is designed to value changes in the water quality of water bodies in Michigan’s lower peninsula, we begin by summarizing the water quality indices. Because the region includes thousands of inland lakes and many miles of rivers, streams, and Great Lakes’ shorelines, it is not feasible to focus on individual water bodies. Instead, existing conditions and proposed water quality changes are described in terms of their impact on water quality at a regional level. For inland water bodies, we use average water quality within 8-Digit Hydrological Units (HUC8s), with 39 such HUC8s in Michigan’s lower peninsula. For Great Lakes shorelines, the lower peninsula shoreline of Michigan is divided into 25 regions (*SI Appendix*, Fig. S8). In all cases, the changes in the indices under the plan come from our experimental design and are applied to baseline to create with and without maps for respondents to review before voting (See *SI Appendix* *f*or an in-depth discussion of all the indices).

To construct the baseline (i.e., status quo or current) levels for each index used in the survey, we sought to distinguish key freshwater ecosystem services related to waterbodies that might be affected by nonpoint source nutrient loads, particularly phosphorus. To capture the potentially distinct effects of water quality change on recreation fishing versus swimming, wading, and boating, we use multiple water quality metrics that break out into 1) an index that focuses on non-fishing water-based recreation (WCS), 2) a recreational game fishing index (FBS), and 3) a nonrecreational index related to biological condition of general aquatic wildlife (our WLS).

FBS indices were formed separately for the Great Lakes regions and the HUC8 inland waterways. For lakes and river and steams, gamefish abundance was estimated for individual waterbodies and then aggregated to HUC8 levels using ecological production functions explicitly tied to phosphorus (11, 35). For Great Lakes HUCs, state creel data on catch rates were used (36). To form a single-valued fish index, the biomass predictions by species were weighted using angling preference weights for species from existing recreation demand models (35–37), an approach based on empirical studies rather than expert judgment.

The WCS is designed to capture the impact water quality has on nonfishing recreation such as boating and swimming and is also explicitly linked to phosphorus. Through subindices, it reflects two potential water quality problems: 1) algal blooms stemming from excess phosphorus and 2) fecal coliform, which can lead to beach closures. These metrics were estimated for individual waterbodies and aggregated to HUC8s.

The WLS scores per HUC8 were based on some biological condition and ecosystem assessment data (38) as well as expert judgment honed through years of expertise within the team doing ecological assessments and studies in these watersheds, which were leveraged to create the baseline maps. Across the two contingent valuation treatments, the underly WLS indices are the same to isolate the effects of combined versus separated recreational indices.

In developing the valuation experiment and survey instrument, we follow guidance in the literature (39) and specifically used the contingent valuation study of Bishop et al. (40) to the extent possible in a self-administered internet survey. We used an iterative design process for qualitative research to test and revise the instrument during which a series of 21 one-on-one cognitive interviews were conducted to test and fine-tune the survey (39, 41). Several pretest pilot surveys were conducted using samples of individuals recruited from Amazon’s Mechanical Turk (MTurk). Both index versions were tested and modified between each of the four pilot waves and with 801 respondents. Results from the pretesting pilot were helpful both in terms of further refining the survey and providing initial parameter estimate priors for the experimental design of scenarios.

The full-scale survey was implemented in six waves (*SI Appendix*, Table S1 and Fig. S2). After each wave of the full survey, parameters were estimated and used to update the experimental design to further increase efficiency of the generated policy scenarios. The specifics of the policy scenario were varied across survey respondents in terms of changes in individual water quality indices, the cost to the household, the spatial scope of the changes, and the number of water quality indices used to describe the policy, i.e., two-index (WQI and WLS) versus three-index (WCS, FBS, and WLS). Table 1 shows the range of levels used for changes in the variables.

**Table 1.** Design attribute levels for the range of changes in quality and baseline quality that appeared across the 30 experimentally designed scenarios

Variable	Description	Levels (#)	Range of changes	Baseline with no policy^†^
Cost	One-time cost (via income tax)	5	$45 to $965	0
ΔWLS	Wildlife score (WLS)	5	0 to 25	57
ΔWCS	Water contact score (WCS)	4	0 to 20	65
ΔFBS	Recreational fishing score (FBS)	5	−5 to 20	65
ΔWQI^*^	Water quality index (WQI)	14	−2 to 21	65

^*^WQI is not a design variable; the function WQI (WCS, FBS) is computed from the design changes in FBS and WCS.

^†^Baseline values are the averages of the mapped HUC8 levels for Michigan’s lower peninsula.

Changes to the WQI were computed using the designed changes to WCS and FBS as inputs in the functional form of WQI. Program costs, communicated as a one-time household tax, were allowed to take on one of five values. The sample was randomly split between those receiving water quality information using two indices versus those receiving three indices. The experimental design used 30 versions of the question scenario that varied cost and water quality changes for the scenario a respondent saw. Across respondents, the survey randomized the scenario shown and the index treatment (*SI Appendix*, section 4.2). The experimental design of the 30 scenarios was done using the NGene design platform to achieve sufficient independent variation across respondents to statistically identify (i.e., characterize) the separate parameters for each WQI and cost.

To maintain comparability across the treatments, the 30 values for changes in the WQI were computed using the 30 versions of WCS and FBS within the experimental design.

**Fig. 1.**
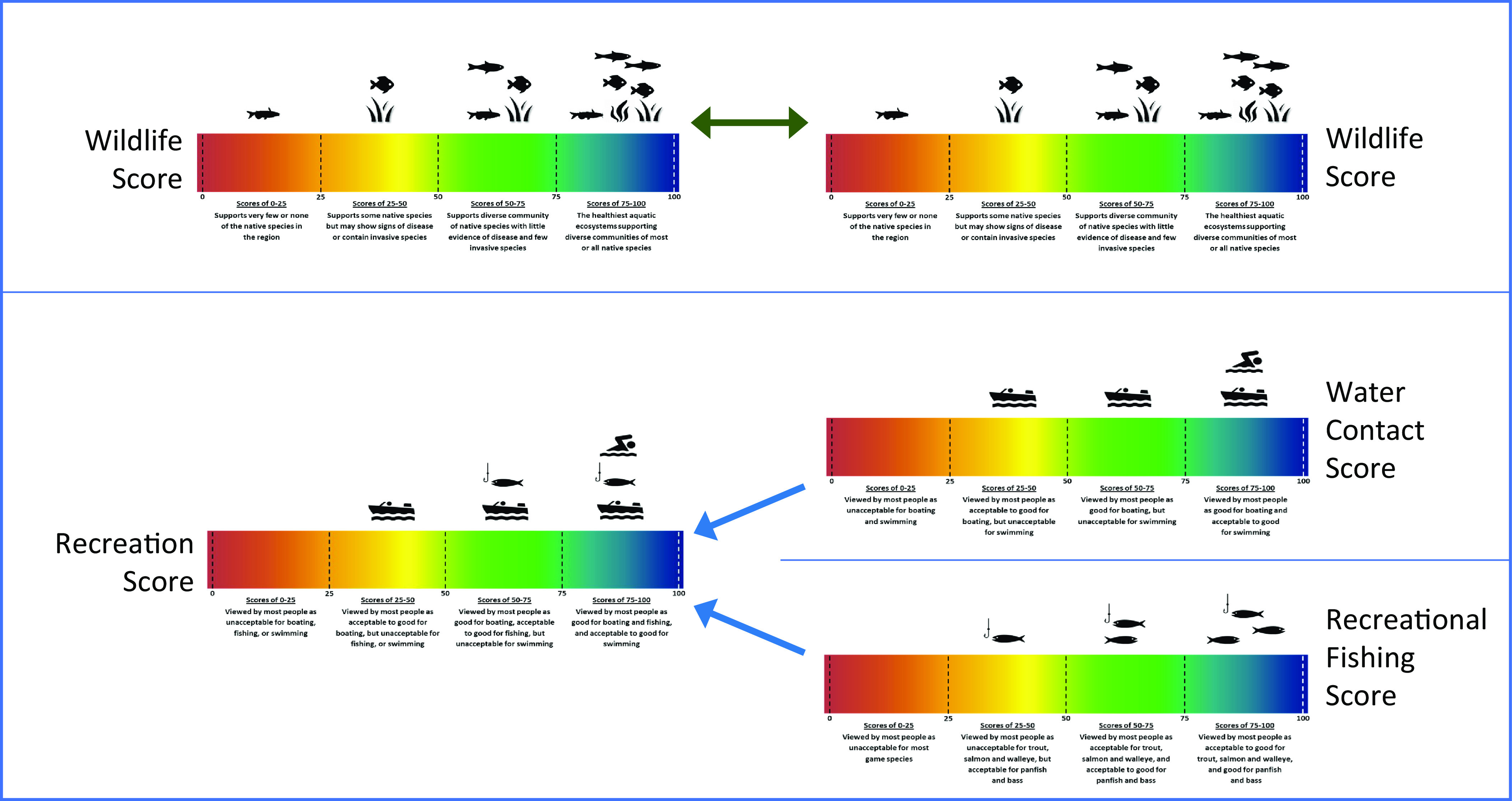
Water quality score scales for the two-index and three-index treatments.

The contingent valuation survey itself was implemented on Qualtrics’ internet panel of Michigan respondents. The survey valuation section began with a brief introduction to water quality and the water quality indices used to characterize both current water quality conditions and the changes that would result from a proposed policy scenario. For each index, respondents were presented information including compact text descriptions and bullets; an image using a color bar and a 100-point scale with quadrants labeled with simple descriptions akin to a ladder; a color-coded map of the spatial distribution of baseline water quality across HUC8s; and interactive questions to promote engagement with the information. Fig. 1 presents a summary of the various color bar scales and quadrant descriptions for the indices used in the two versus three index treatments (see *SI Appendix*, Figs. S3–S6 for the separate images).

**Fig. 2.**
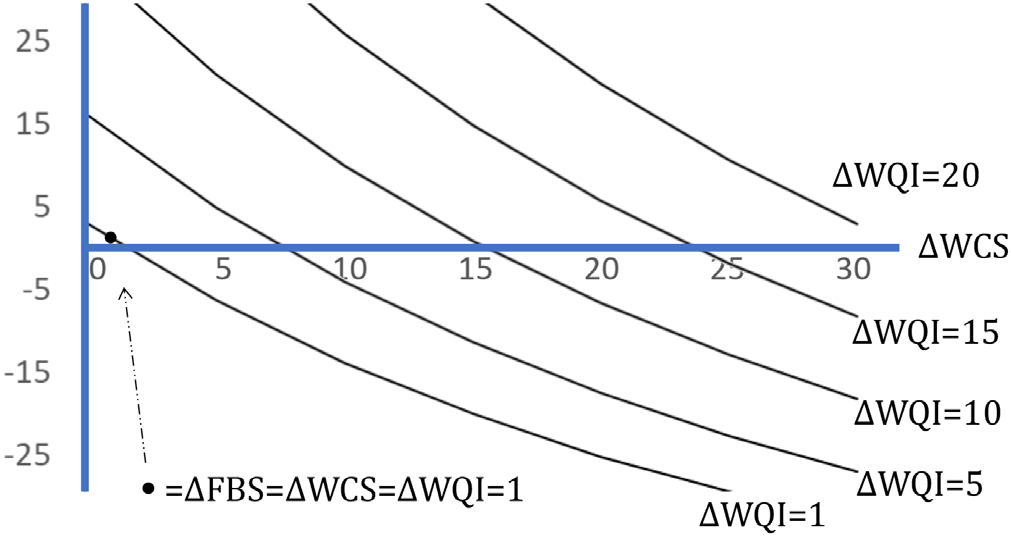
Maps of baseline water quality scores across HUC8s shown in the survey. Figure Notes: WQI and WLS were for two-index treatment, and WQI, WCS, and WLS were used for three-index treatment. The two-index baseline uses a WQI that was derived from WCS and FBS using the function WQI (WCS, FBS). The figure combines images for convenience; respondents saw them sequentially with other material about each index (*SI Appendix*, section 4.3).

 Fig. 2 shows all four baseline quality maps used in the survey: WQI and WLS in the two-index treatment and WCS, FBS, and WLS in the three-index treatment. Maps were presented to respondents sequentially in conjunction with descriptive text, questions stimulating interaction with the information, and the image for that index scale (Fig. 2 and *SI Appendix*, Fig. S7). From Fig. 2, one can see that the WQI colors are between the higher FBS scores and the lower WCS scores, which reflects the construction of WQI as a function of WCS and FBS. Moreover, the index is better for FBS than for WCS in policy-relevant areas where WCS is more degraded such as southeast Michigan.

Next, the survey presents details of a single policy to be voted on in a dichotomous choice referendum, which has desirable incentive properties (42). The plan is presented in terms of statewide changes to each WQI and another set of maps illustrating the corresponding changes for each index. Household income is elicited before the plan’s cost is presented to enhance realism of an income tax payment à la Bishop et al. (40). Respondents are told that the plan they see is “the only plan under consideration,” their answers will “be shared with policy-makers that may use them to implement the plan,” and reiterating that they will have to pay the one-time cost if the plan is implemented, which together reinforce the consequentiality of their vote in this one-shot referendum (42). Respondents are also given example reasons that some people may vote for or against the plan to potentially reduce possible social desirability bias. The plan is again summarized in a single page with the table summarizing the changes and cost. For the core contingent valuation question, the survey then asked participants to vote in a dichotomous choice referendum considering a single policy impacting the average levels for each index and the cost to their household. Following the vote, respondents are asked to provide comments on their reasons for their vote, which are assessed for validity and fraud.^†^ Finally, attitude scales were used to measure various factors that may have influenced their choice.

## Results

In total, the Qualtrics survey panel yielded 1,718 completed surveys for the analysis. Demographic variables and response numbers per wave were well balanced across the two randomly assigned treatments (*SI Appendix*, Tables S1 and S2). The series of validity checks regarding survey responses included the extent to which respondents believe the survey is consequential and neutral in its presentation (43). Neutrality was assessed in the validity question before the attitude scales by asking whether respondents felt that the study “pushed” them to vote for or against the policy; 80% said that it let them make up their own mind with no significant difference across treatments. For the remaining validity questions, we assess disagreement by respondents who “disagreed” or “somewhat disagreed” with a particular statement. For the policy or cost consequentiality statements, disagreement was 11% for policy and 10% for cost. For water quality, those with disagreement about caring for various measures of water quality were in the minority, with disagreement being 5% for water quality generally, 4% for WLS, 5% for WCS, 8% for WQI, and 15% for FBS. None of the above measures differed significantly for the two- and three-index treatments (though WQI appears only with two-index versions and WCS and FBS are only in three-index versions). The results are consistent with theory and desired outcomes for validity of a contingent valuation survey (39, 43).

The econometric analysis of the survey referendum uses a logit model of the discrete choice to vote for or against the proposed policy scenarios. The choice is driven by the difference in respondent’s utility with and without the policy, where the utility is a function of the policy changes in water quality attributes and the policy cost to the household (see *SI Appendix* *f*or further estimation details and results). Tests show that the results are robust to a variety of alternative specifications for the respondent utility for the policy (*SI Appendix*, Tables S3–S5). In both treatments, all the parameters of interest are significant at the 5% level. Cost has a negative effect on votes, and the water quality parameters have positive effects on votes, i.e., plans with lower costs or higher quality changes increase the share of respondents voting for the plan. In the three-index model, the effect of a small change in WCS is larger than for FBS, with the effect of a small change in the WQI in the two-index model lying between those of its respective subindices. A key finding that bears on benefit estimation is that when a marginal change in only one underlying subindex is mapped through the WQI function in the model with the WQI alone, the value for changes in WCS is roughly 50% smaller than the respective marginal value in the model with FBS and WCS modeled separately (*SI Appendix*, Tables S5 and S6).

**Fig. 3.**
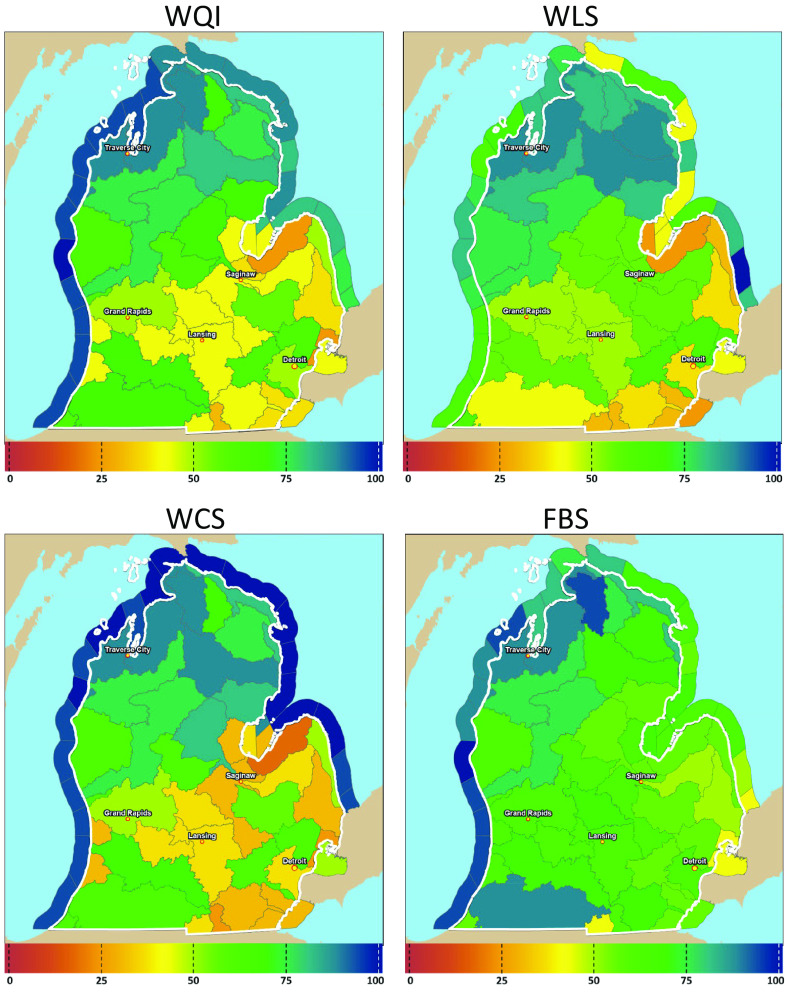
Level curves for the changes in FBS (denoted by ΔFBS) and changes in WCS (ΔWCS) that yield the same ΔWQI from the function for WQI (WCS, FBS). Figure Notes: As the change in WQI increases, the level curves increase to the northeast. The bold point, ●, shows where the changes in each index equal one. The vertical scale is compressed for space. Derivations are reported in *SI Appendix*, section 1.4 and Table S7.

One of the primary reasons for dividing the WQI into component parts and presenting them separately to survey respondents is that policies may cause the individual indices (i.e., different ecosystem services) to move differentially or even in opposite directions. These individual movements would be masked if only the composite WQI were presented. For example, one could envision improvements in the WCS for a waterbody that would at the same time lead to decrements to the FBS. For example, it is possible to improve WCS in a way that harms FBS through decreases in phosphorus that increase water clarity yet adversely affect P-limited aquatic vegetation for key gamefish such as walleye and bass (11). To illustrate this issue, consider specifying a policy that changes WQI by ΔWQI. This change can be achieved by an infinite number of combinations of changes to FBS and WCS, but for any change in WCS (ΔWCS), the corresponding change in FBS (ΔFBS) can be solved when holding the WQI constant. Fig. 3 plots some combinations that can yield the same ΔWQI, i.e., it shows the “level curves” for WQI (WCS, FBS). As the change in the WQI increases, the level curves in Fig. 3 increase to the northeast. Note that Fig. 3 (as well as some subsequent graphs) plots changes for ΔWQI that are all within the range of the experimental design, but some of the combinations of changes to FBS and WCS that create each level curve lie outside their range in the design for the sample data.

**Fig. 4.**
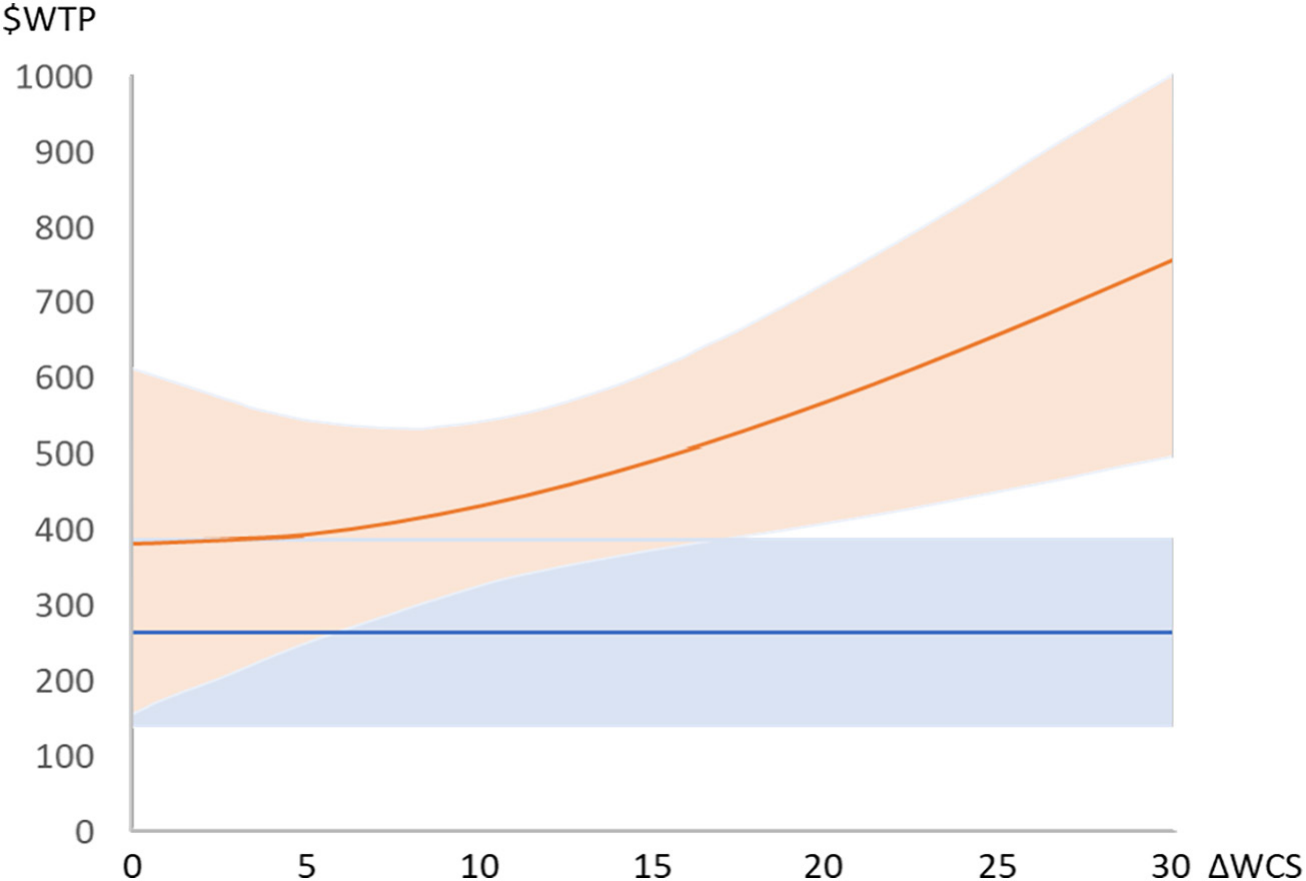
Willingness to pay ($WTP) for service changes for the combination of ΔFBS and ΔWCS that yield ΔWQI = 10 in the formula for WQI. Figure Notes: Shaded areas represent 90% CIs for either the two or three index versions (but are not the same as the test of difference). The difference between the two WTP lines is significant at 10% for all values of ΔWCS from 10 and larger. The corresponding ΔFBS are in Fig. 3. For example, ΔFBS = 0.92 and ΔWCS = 15 give ΔWQI = 10 and result in WTP = $263 in the two-index model and WTP = $491 in the three-index model (*SI Appendix*, Table S8).

To illustrate the implication of using the WQI for valuation of underlying changes in the subindices FBS and WCS, Fig. 4 plots the economic benefit of a change of WQI = 10 using the two-index and three-index models for a nonmarginal change. The lines represent sample average WTP, and shaded areas are CIs. When directly using the two-index model to value ΔWQI = 10 (the blue line), the value is always smaller than when they are valued using the three-index model having values for the various combinations of ΔFBS and ΔWCS that yield ΔWQI = 10. The difference between the valuations from the two models increases as the WCS changes become larger, even at a cost of a reduction in FBS—a shape driven by the fact that respondents valued a change in the WCS more than a change in FBS.^‡^ In Fig. 4, all combinations with ΔWCS of 15 or higher give economic values that are significantly different at 5% or better for the two and three index treatments.

**Fig. 5.**
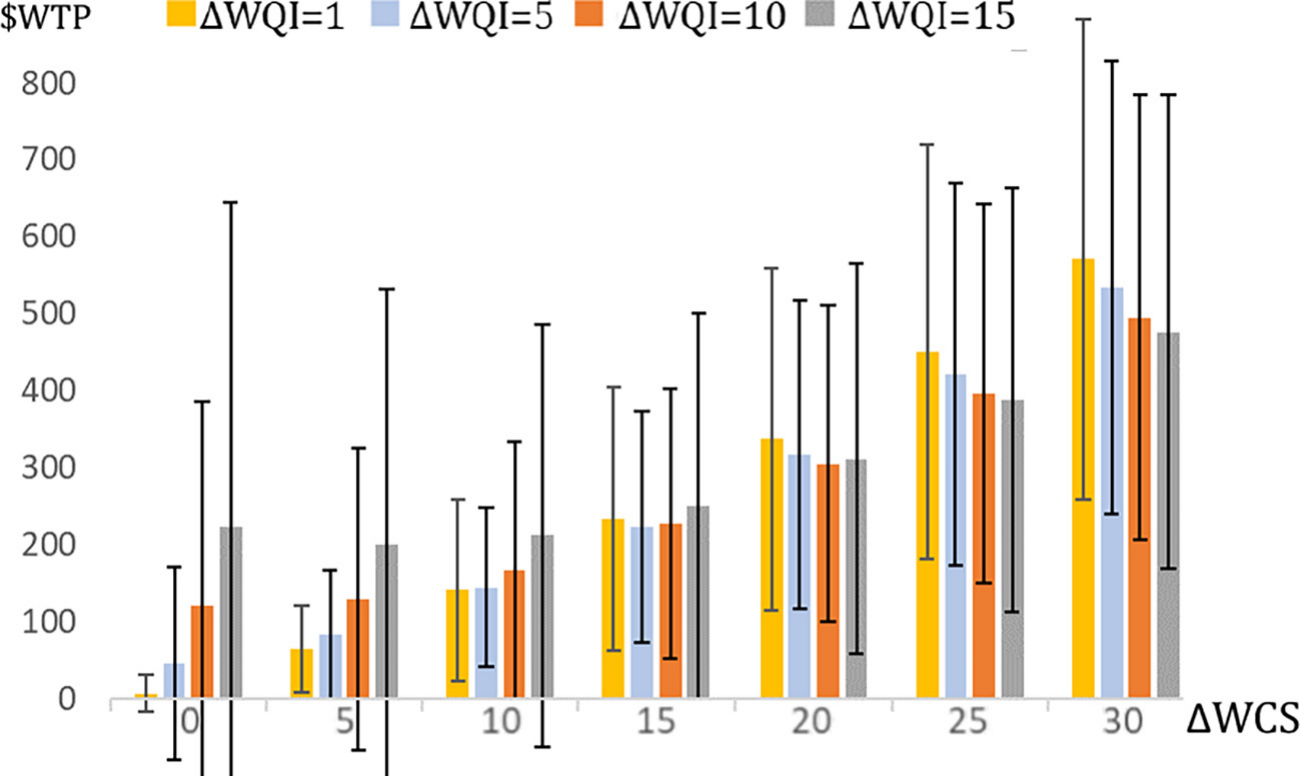
Differences in WTP ($WTP) between three-index and two-index treatments for combinations of ΔFBS and ΔWCS that yield various ΔWQI in the formula for WQI. Figure Notes: For example, the dark orange bars for ΔWQI = 10 represent the WTP differences between the lines in Fig. 4. The bar values are positive since the three-index WTP is always larger than in the two-index. Error bars are 90% CIs for the predicted WTP$ (*SI Appendix*, Table S8).

Across a broad range of changes in the WQI, the results show that when using the three-index model for valuation of the corresponding combinations of ΔFBS and ΔWCS, the three-index models produce larger values than when the same subindex combinations are valued through the WQI alone. Fig. 5 depicts this result using bars to represent the differences in valuation to the three-index and two-index treatments for several alternative values of ΔWQI. The individual bars represent four possible ΔWQI, and the sets of bars represent levels of ΔWCS consistent with any ΔWQI (see *SI Appendix*, Table S7 for corresponding FBS). The CIs around the bars show that most of the time, the three-index valuations are significantly larger and increasingly so with small ΔWQI or larger ΔWCS.

## Discussion

Many benefit–cost analyses and policy-relevant studies from the United States are done using valuation models or original studies based on the WQI or a water quality ladder (6, 14). Moreover, many recent Federal water quality regulations also do not pass a BCA (13). One challenge may be that the traditional WQI aggregates all facets of water quality into a single metric. As noted above, while this simplifies the task of characterizing a given water body, it potentially hides economically important information regarding the mix of ecosystem services that can be provided. Yet for water quality and other environmental changes, the resulting effects on ecosystem services often invoke trade-offs in some of the underlying services even when the overall changes are beneficial (1), just as benefit–cost analyses of ecosystem changes that do not consider distributional effects can mask underly losses to marginalized populations (44). Here, we address the question of whether estimated recreational water quality valuations differ when survey respondents are given the more detailed characterizations of water quality versus one that aggregates them. Toward this end, we developed a split-sample contingent valuation study in which, for half of the sample, water quality related to recreation is described using two recreational indices: a FBS and a WCS along with a WLS for biological condition. For the other half of the sample, the FBS and WCS subindices are aggregated into a single WQI. Our study confirms our hypothesis that for some underlying trade-offs between key ecosystem services, it matters whether those services are valued by combining ecosystem services within a single index or separating the services in separate indices. We also find that when a marginal change in WCS is mapped through the WQI function in the model with WQI alone, the value is significantly smaller than the respective marginal value in the model with WCS modeled separately, which is relevant for valuation of recreational services.

We note that our study was specifically designed to test the two versus three index treatments rather than for a BCA for the general population. This is especially important since other studies suggest that there can be important differences between total values when random samples of the general population are compared to convenience internet samples such as ours (39, 45). However, for a rough illustration of the magnitude of some of the differences we found, we apply the results to Michigan. Also, note that the magnitudes of the valuation of water quality from our models that are reported above are present values since we used a one-time income tax as the payment for the plan. To translate the results into an annual value into perpetuity, we need to use a discount rate. The US Office of Management and Budget guidance to Federal agencies is to use between 3% and 7% for regulatory analysis such as BCA^§^ [see also Horsch et al. (46)]. Using 3%, the annual value of a change in the WQI of 10 due to changes in both WCS and FBS is $7.90 per adult per year in the WQI model and $12.93 per adult per year in the model handling FBS and WCS separately, and in all cases, using shorter timeframes to convert the present values would give larger annual values.^¶^

Recognizing that our sample need not represent the population, we nevertheless illustrate the aggregate statewide estimates as if the results are applicable to the population of Michigan. A water quality change of 10 in all subindices translates to $102 million per year in Michigan, whereas it would be $63 million if a model done solely with a WQI was used. For convenience, these illustrations all hold WLS constant, even though it too would likely change. However, if the change in WCS was 20, yet the overall change in WQI stayed at 10, our preferred model would value that change at $135 million per year in Michigan, which is over twice or $73 million more in Michigan than if the changes were mapped through a single WQI approach. It is not hard to imagine that there would be some policies for which the differences would be sufficient to sway the balance of benefits and costs.

Future research could explore whether there are similar differences when moving past the WQI in other locations and whether these differences would change regulatory analyses akin to how Walsh and Wheeler (14) found that aggregation within a WQI, when applied to actual Federal benefit–cost analyses, could change the outcomes. Future work could also focus more survey effort toward understanding whether respondents are inferring correlations among biological condition and the other indices when they see a recreational WQI versus separate recreational indices for WCS and FBS. In the existing research, our next steps will explicitly link the WLS to ecological production functions which depends on nutrients affecting ecosystem services as we have linked our FBS and WCS to pollution concentrations. Doing so would allow tests to incorporate more complete benefit estimates since many pollution changes will also affect the nongamefish aquatic organisms.

## Supplementary Material

Appendix 01 (PDF)

## Data Availability

Data have been deposited in Zenodo (URL: https://zenodo.org/record/7693585#.ZAYe-i-B3Pw, DOI: 10.5281/zenodo.7693585) (47).

## Footnotes

^†^Fraudulent and robotic responses in paid online surveys have spurred the development of enhanced fraud-detection by vendors and by researchers. Fraud was not detected in this survey, but it was detected in some of our subsequent surveys and has arisen for other valuation efforts (48).

^‡^The finding is consistent with the data showing only 20% of the respondents fish (*SI Appendix*, Table S2).

^§^See Office of Management and Budget, (OMB), “Circular A-4.” https://obamawhitehouse.archives.gov/omb/circulars_a004_a-4/.

^¶^If instead we use a 7% rate, values and difference are $18.43/adult/y and $30.16/adult/y respectively.

## References

[1] J. H. Goldstein , Integrating ecosystem-service tradeoffs into land-use decisions. Proc. Natl. Acad. Sci. U.S.A. **109**, 7565–7570 (2012). 22529388 10.1073/pnas.1201040109 PMC3358905

[2] C. Raudsepp-Hearne, G. D. Peterson, E. M. Bennett, Ecosystem service bundles for analyzing tradeoffs in diverse landscapes. Proc. Natl. Acad. Sci. U.S.A. **107**, 5242–5247 (2010). 20194739 10.1073/pnas.0907284107 PMC2841950

[3] R. Naidoo , Global mapping of ecosystem services and conservation priorities. Proc. Natl. Acad. Sci. U.S.A. **105**, 9495–9500 (2008). 18621701 10.1073/pnas.0707823105 PMC2474481

[4] R. Naidoo, W. L. Adamowicz, Economic benefits of biodiversity exceed costs of conservation at an African rainforest reserve. Proc. Natl. Acad. Sci. U.S.A. **102**, 16712–16716 (2005). 16267131 10.1073/pnas.0508036102 PMC1283836

[5] K. J. Arrow , Is there a role for benefit-cost analysis in environmental, health, and safety regulation? Science **272**, 221–222 (1996). 8602504 10.1126/science.272.5259.221

[6] C. Griffiths , Environmental Protection Agency valuation of surface water quality improvements. Rev. Environ. Econ. Policy **6**, 130–146 (2012).

[7] R. Carson, R. Mitchell, The value of clean water: The public’s willingness to pay for boatable, fishable, and swimmable quality water. Water Resour. Res. **29**, 2445–2454 (1993).

[8] N. McClelland, Water Quality Index Application in the Kansas River Basin. EPA907/9-74-001, February. (1974). https://nepis.epa.gov/Exe/ZyPURL.cgi?Dockey=20008TH7.txt.

[9] W. J. Vaughan, “The water quality ladder” in The Use of Contingent Valuation Data for Benefit Cost Analysis in Water Pollution Control, R. C. Mitchell, R. T. Carson, B. Appendix, Eds. (U.S. EPA Office of Policy and Evaluation, Washington, DC, 1986), p. CR-810224-02.

[10] G. Van Houtven, J. Powers, S. K. Pattanayak, Valuing water quality improvements in the United States using meta-analysis: Is the glass half-full or half-empty for national policy analysis? Resour. Energy Econ. **29**, 206–228 (2007).

[11] P. Esselman, R. J. Stevenson, F. Lupi, C. R. Riseng, M. J. Wiley, Landscape prediction and mapping of game fish biomass, an ecosystem service of Michigan rivers. North Am. J. Fish Management **35**, 302–320 (2015).

[12] P. C. Jacobson, G. J. A. Hansen, B. J. Bethke, T. K. Cross, Disentangling the effects of a century of eutrophication and climate warming on freshwater lake fish assemblages. PLOS One **12**, e0182667 (2017). 28777816 10.1371/journal.pone.0182667 PMC5544199

[13] D. Keiser, C. Kling, J. Shapiro, The low but uncertain measured benefits of US water quality policy. Proc. Natl. Acad. Sci. U.S.A. **116**, 5262–5269 (2019). 30297391 10.1073/pnas.1802870115 PMC6431143

[14] P. J. Walsh, W. Wheeler, Water quality indices and benefit-cost analysis. J. Benefit-Cost Anal. **4**, 81–105 (2013).

[15] R. K. Horton, An index-number system for rating water quality. J. Water Pollut. Control Federation **37**, 300–305 (1965).

[16] U.S. Environmental Protection Agency, Environmental Impact and Benefits Assessment for Final Effluent Guidelines and Standards for the Construction and Development Category (Office of Water, November, 2009).

[17] R. J. Johnston, E. Besedin, R. Stapler, Enhanced geospatial validity for meta-analysis and environmental benefit transfer: An application to water quality improvements. Environ. Resource Econ. **68**, 343–375 (2017).

[18] R. J. Johnston, D. M. Bauer, Using meta-analysis for large-scale ecosystem service valuation: Progress, prospects, and challenges. Agricultural and Resource Econ. Rev. **49**, 23–63 (2020).

[19] J. Corona , An integrated assessment model for valuing water quality changes in the United States. Land Econ. **96**, 478–492 (2020). 34017148 10.3368/wple.96.4.478 PMC8128698

[20] J. A. Downing, S. B. Watson, E. McCauley, Predicting Cyanobacteria dominance in lakes. Canadian J. Fisheries and Aquatic Sci. **58**, 1905–1908 (2001).

[21] C. M. Riseng, M. J. Wiley, R. J. Stevenson, Hydrologic disturbance and nutrient effects on benthic community structure in midwestern US streams: a covariance structure analysis. J. North Am. Benthol. Soc. **23**, 309–326 (2004).

[22] S. T. Rier, R. J. Stevenson, Response of periphytic algae to gradients in nitrogen and phosphorus in streamside mesocosms. Hydrobiologia **561**, 131–147 (2006).

[23] P. A. Soranno , A framework for developing ecosystem-specific nutrient criteria: Integrating biological thresholds with predictive modeling. Limnol. Oceanogr. **53**, 773–787 (2008).

[24] R. J. Stevenson, B. E. Hill, A. T. Herlihy, L. L. Yuan, S. B. Norton, Algal-P relationships, thresholds, and frequency distributions guide nutrient criterion development. J. North Am. Benthol. Soc. **27**, 783–799 (2008).

[25] J. A. Downing, E. McCauley, The nitrogen - phosphorus relationship in lakes. Limnol. Oceanogr. **37**, 936–945 (1992).

[26] E. Jeppesen , Does the impact of nutrients on the biological structure and function of brackish and freshwater lakes differ? Hydrobiologia **275**, 15–30 (1994).

[27] T. G. Zorn, A. J. Nuhfer, Influences on brown trout and brook trout population dynamics in a michigan river. Trans. Am. Fish. Soc. **136**, 691–705 (2007).

[28] Y.-C. Kao, S. Adlerstein, E. Rutherford, The relative impacts of nutrient loads and invasive species on a Great Lakes food web: An Ecopath with Ecosim analysis. J. Great Lakes Res. **40**, 35–52 (2014).

[29] Y.-C. Kao, S. A. Adlerstein, E. S. Rutherford, Assessment of top-down and bottom-up controls on the collapse of alewives (Alosa pseudoharengus) in Lake Huron. Ecosystems **19**, 803–831 (2016).

[30] R. J. Miltner, E. T. Rankin, Primary nutrients and the biotic integrity of rivers and streams. Freshwater Biol. **40**, 145–58 (1998).

[31] B. L. Keeler , Linking water quality and well-being for improved assessment and valuation of ecosystem services. Proc. Natl. Acad. Sci. U.S.A. **109**, 18619–18624 (2012). 23091018 10.1073/pnas.1215991109 PMC3494932

[32] S. Hime, I. J. Bateman, P. Posen, M. Hutchins, A transferable water quality ladder for conveying use and ecological information within public surveys. CSERGE Working Paper EDM 09-01 - Centre for Social and Economic Research on the Global Environment (**1**), 1–36 (University of East Anglia, Norwich, UK, 2009).

[33] R. Hill , Valuing Aquatic Ecosystem Health at a National Scale: Modeling Biological Indicators Across Space and Time (U.S. Environmental Protection Agency, National Center for Environmental Economics, Working Paper 20–04, November 2020, 2020).

[34] U.S. Environmental Protection Agency, A practitioner’s guide to the biological condition gradient: A framework to describe incremental change in aquatic ecosystems (Office of Water, Washington DC, EPA 842-R-16-001 2016).

[35] F. Lupi , Linking agricultural nutrient pollution to the value of freshwater ecosystem services. Land Econ. **96**, 493–509 (2020).

[36] R. Melstrom, F. Lupi, Valuing recreational fishing in the great Lakes. North Am. J. Fish. Manage. **33**, 1184–1193 (2013).

[37] R. Melstrom, F. Lupi, P. Esselman, R. J. Stevenson, Valuing recreational fishing quality at rivers and streams. Water Resources Res. **51**, 140–150 (2015).

[38] J. D. Allan , Joint analysis of stressors and ecosystem services to enhance restoration effectiveness. Proc. Natl. Acad. Sci. U.S.A. **110**, 372–377 (2013). 23248308 10.1073/pnas.1213841110 PMC3538252

[39] R. J. Johnston , Contemporary guidance for stated preference studies. J. Assoc. Environ. Resource Econ. **4**, 319–405 (2017).

[40] R. C. Bishop , Putting a value on injuries to natural assets: The BP oil spill. Science **356**, 253–254 (2017). 28428387 10.1126/science.aam8124

[41] M. Kaplowitz, F. Lupi, J. Hoehn, Multiple-methods for developing and evaluating a stated preference survey for valuing wetland ecosystems. Chpt. 24 In Questionnaire Development, Evaluation, and Testing Methods, S. Presser , eds. (Wiley, New Jersey, 2004). pp. 503–524.

[42] R. Carson, T. Groves, Incentive and informational properties of preference questions. Environ. Resource Econ. **37**, 181–210 (2007).

[43] R. Bishop, K. Boyle, Reliability and validity in nonmarket valuation. Environ. Resource Econ. **72**, 559–82 (2019).

[44] T. Daw , Evaluating taboo trade-offs in ecosystems services and human well-being. Proc. Natl. Acad. Sci. U.S.A. **112**, 6949–6954 (2015). 26038547 10.1073/pnas.1414900112 PMC4460479

[45] K. Sandstrom, F. Lupi, H. Kim, J. A. Herriges, Comparing water quality valuation across probability and non-probability samples. Agricultural Economics and Public Perspectives, In press, Available at: https://sites.google.com/msu.edu/franklupi/home.

[46] E. Horsch , Discounting in natural resource damage assessment. J. Benefit-Cost Anal. 1–21 (2022), 10.1017/bca.2022.24.

[47] F. Lupi , data for Disentangling water quality indices to enhance the valuation of divergent ecosystem services PNAS [Data set]. In Proceedings of the National Academy of Science of USA. Zenodo. 10.5281/zenodo.7693585. PMC10160960 37094116

[48] J. Johnston, F. Lupi , Do you know who’s answering your survey? Expanding threats to the integrity of online panel data in environmental and resource economics. Association of Environmental and Resource Economists (AERE) Summer Conference (2021).

