# Supplementary material for: Getting off the ladder: Disentangling water quality indices to enhance the valuation of divergent ecosystem services
Source: Proc Natl Acad Sci U S A. 2023 Apr 24;120(18):e2120261120. doi: 10.1073/pnas.2120261120 (PMC10160960; doi:10.1073/pnas.2120261120)
Supplement: Supplementary file 1 — Appendix 01 (PDF) [file pnas.2120261120.sapp.pdf]

Supplemental Information

Sections

S1. Water quality metrics
S2. Using WQI index for changes in FBS and WCS
S3. Econometric details
S4. Additional survey details
S5. Estimation results, alternative specifications, and valuation
S6. References

Supplemental Tables

Table S1: Distribution of survey completes by round and numbers of indices used

Table S2: Treatment balance of demographics

Table S3: Model results comparing 2- and 3-Index with and without demographics.

Table S4: Model results comparing 2- and 3-Index with linear and ln quality.

Table S5: Model results comparing marginal values with and without demographics.

Table S6: Model results comparing tests of constraints on marginal values implied by how *WQI* would work in practice when a sub-index changes.

Table S7: Level curves of ΔWCS and ΔFBS for various ΔWQI

Table S8: Differences in willingness to pay ($WTP) for combinations of ΔFBS and ΔWCS yielding various ΔWQI in the formula for *WQI*.

Supplemental Figures

Figure S1: A classic Water Quality Ladder Wildlife (from Jeon et al. 2005)

Figure S2: Survey development, testing, and implementation timeline

Figure S3: Wildlife score (WLS) used in both 2- and 3-index treatments

Figure S4: Water recreation score (*WQI*) used in 2-index treatment

Figure S5: Water contact score (*WCS*) used in 3-index treatment

Figure S6: Fish biomass score (*FBS*) used in 3-index treatment

Figure S7: Information example: Baseline *WQI* and an interaction question

Figure S8: Information example: Watershed map to illustrate idea

Figure S9: Information example: How the plan affects water quality

Figure S10: Plan payment information and income elicitation

Figure S11: Information example: Reasons to vote for and against

Figure S12: Plan information reminder and voting question

Figure S13: Summary of content analysis of open-ended comments for reasons for vote

Figure S14: Votes for the plan tend to decline with higher payment

Figure S15: Marginal values of WCS and FBS in two versus three index models

S1. Water quality metrics

1.0 Additional background on WQI

In McClelland's first survey, a panel of experts were asked to designate which of thirty-five water quality parameters should be included in an index of water quality and then to rate (on a five-point scale) those that should be included "...according to their significance to overall water quality." (McClelland, 1974, p. 8), which was used to narrow the set of water quality measures to be included in the index down to eleven parameters. A subsequent survey used expert elicitation to obtain curves on a scale from 0 to 100 to represent water quality for each index parameter (McClelland, 1974, p. 9), which were averaged across responding experts, this effectively produced a sub-index for each water quality measure, indicating how changes in each water quality measure individually impacted overall water quality. Finally, survey respondents were asked to rate the remaining eleven water quality parameters on a 5-point scale in terms of their significance to water quality. These ratings were used to develop weights assigned to each water quality parameter in the multiplicative index that became McClelland's WQI. Specifically, McClelland's WQI is the product of exponentially weighted sub-indices given by:

$$WQI = \prod_{i=1}^{n} q_i^{w_i}$$

where q_i denotes a sub-index for water quality parameter *i* (as determined by the water quality "curves" elicited from the panel of experts); w_i denotes the weight assigned to water quality parameter *i* which sum to one across the *n* sub-indices.

Ultimately, McClelland's index included *n* = 9 factors: dissolved oxygen, fecal coliform, pH, nitrates, phosphates, 5-day BOD, temperature, total solids and turbidity. In subsequent work, the USEPA (2009) narrowed the number of water quality parameters by dropping total solids, pH and temperature, rescaling the remaining weights so that they summed to one. They also replace McClelland's sub-indices for individual water quality parameters, q_i in the equation above, with similar indices developed by Dunnette (1979) and Cude (2001) for the Oregon Water Quality Index (*O*WQI). Vaughan (1986) identified minimally acceptable levels of WQI for each of five designated uses: drinking (WQI=95), swimming (WQI=70), game fishing (WQI=50), rough fishing (WQI=45) and boating (WQI=25) (USEPA, 2009, p. 10-6). These categories, in turn, represented rungs on the now familiar water quality ladder (see *SI Appendix*, Figure S1).

1.1 Fish biomass score (*FBS*)

The Fish Biomass Score (*FBS*): *FBS* indices (depicted in Figure S2) were formed separately for the Great Lakes regions and the HUC8 inland waterways. In the case of the Great Lakes, county catch rates for six species: Chinook, Coho, Lake Trout, Steelhead, Walleye and Perch (Lupi et al. 2020) were matched to Great Lakes shoreline regions in Figure S8. The county average catch rates by species were then combined into a single index (our *FBS*) using preference weights of Melstrom and Lupi (2013), with the index normalized to lie between 40 and 100. In the case of inland waterways, fish indices were formed separately for inland lakes versus rivers and streams for each HUC8. For river segments, species level biomass predictions were obtained from boosted regression trees generated by Peter Esselman (Esselman et al., 2015; Melstrom et al. 2015). Separate estimates were obtained for five species: Brook Trout, Brown Trout, Walleye, Bass, and Panfish. Esselman aggregated his predicted biomasses for each species to a HUC10 level using a length weighted average of the biomass for each site within the HUC10. These were then combined into a single index using the preference weights based on fishing effort and preferences (Melstrom and Lupi, 2013; Melstom et al., 2015; Lupi et al., 2020).[1] HUC8 level indices for rivers (FBS_{rivers}) were obtained as a stream length weighted average of HUC10 indices, with the index normalized to lie between 40 and 100. A parallel process was used to form a HUC8 level fish biomass index for inland lakes (FBS_{lakes}).[2] Finally, the overall *FBS* for each HUC8 was then formed as the geometric mean of FBS_{rivers} and FBS_{lakes}.

1.2 The Water Contact Score (WCS):

The WCS is designed to capture the impact water quality has on non-fishing recreation, e.g., boating, swimming, etc. Through sub-indices, it reflects two potential water quality problems: (1) algae blooms stemming from excess nutrients (total phosphorus, TP), and (2) fecal coliform (FC), which can lead to beach closures.

In constructing the *TP* sub-index, median total phosphorus reading was first computed for each Michigan station in the US Water Quality (USWQ) database. Within each HUC8, median *TP* across stations was then computed. For the Great Lakes beach zones, an adjusted *TP*, TP_g, was formed to reflect differences between offshore and nearshore *TP*

[1] Compared to just using expert judgements, the preference weights have the advantage of being empirically based in the study area and being based on relatively large samples--4,705 for Great Lakes (Melstrom and Lupi 2013), 1,591 for rivers (Melstrom et al. 2015), and 8,245 for lakes (Lupi et al. 2020).

[2] There were two differences in the process for inland lakes relative to that for rivers. First, for inland lakes, there were four fish species considered: Perch, Walleye, Bass, and Panfish. Second, while biomass predictions for rivers were available for all river and stream segments, the inland lake biomass predictions were available only for lakes over 10 acres visited by at least one person in angler surveys and were developed using boosted regression trees in the same manner as in Esselman et al. (2015).

readings. For interior HUC8's $TP_g = TP$. The total phosphorus sub-index SIf_{TP} was then formed using:

$$SIf_{TP} = 100 \cdot 2^{-50 \cdot TP_g \cdot Sh} \tag{S1}$$

where Sh is a "shallowness" indicator ($Sh \in 1,2,3$), with $Sh = 3$ denoting the shallowest water. The inclusion of Sh in the sub-index SI_{TP} was to penalized shallow zones, to reflect the increased potential for algae blooms in shallow water.[3] The final sub-index was formed by truncating SIf_{TP} to lie strictly between 10 and 100, as is the case for all of the historical EPA sub-indices, i.e.,

$$SI_{TP} = max(10, SIf_{TP}) \tag{S2}$$

The sub-index for fecal coliform was formed based on the linkage between median fecal coliform readings at a station and the probability of a beach closure stemming from high levels of *E. coli*. The starting point of the process was to use *E. coli* readings from the USWQ database. These readings were used to form two variables:

- C_s: The probability of closure at station s computed as the percentage of *E. coli* readings at the station exceeding 300.[4]
- FC_s: The median fecal coliform reading for station s.[5]

A linear regression model of C_s as a function of FC_s was then used to form a fitted closure probability ($\hat{C}_s$) for each station in the USWQ database.[6] In converting the station level FC_s to a sub-index, we sought to link FC_s to specific probabilities of closure; e.g., 1 in 20, 1 in 10 etc. A piecewise linear function was constructed with the following nodes:

- SIFC=10 if $\hat{C}_s > 0.20$ (greater than a 1 in 5 predicted chance of closure), roughly the 99*th* percentile;

[3] These zones included near Pilgrim (zone 7), Saginaw Bay (zones 17, 18 and 29), Lake St. Claire (zone 24) and Lake Erie (zone 25).

[4] See "About Beach Monitoring in Michigan" for more about precisely how beach closures are determined in the state (http://www.michigan.gov/deq/0,4561,7-135-3313 3681 3686 3730-11005–,00.html).

[5] The USWQ data provides E. coli readings at stations. These were converted to fecal coliform readings using $FC = (E.\ coli/0.53)^{1/1.03}$

[6] Quadratic and trimmed regression models were also explored but did not yield significant improvements in terms of fit.

- SIFC=20 if $\hat{C}_s = 0.10$ (a 1 in 10 predicted chance of closure), roughly the 95th percentile;
- SIFC=40 if $\hat{C}_s = 0.05$ (a 1 in 20 predicted chance of closure), roughly the 80th percentile;
- SIFC=60 if $\hat{C}_s = 0.02$ (a 1 in 50 predicted chance of closure), roughly the 60th percentile;
- SIFC=80 if $\hat{C}_s = 0.01$ (a 1 in 100 predicted chance of closure), roughly the 50th percentile;
- SIFC=100 if $\hat{C}_s = 0$.

The corresponding equation for the *FC* sub-index becomes:

$$SIFC = \begin{cases} 100 & \hat{C}_s = 0 \\ 100 - 2000\hat{C}_s & 0 < \hat{C}_s \leq 0.02 \\ 60 - \frac{2000}{3}(\hat{C}_s - 0.02) & 0.02 < \hat{C}_s \leq 0.05 \\ 40 - 400(\hat{C}_s - 0.05) & 0.05 < \hat{C}_s \leq 0.10 \\ 20 - 200(\hat{C}_s - 0.10) & 0.10 < \hat{C}_s \leq 0.20 \\ 10 & \hat{C}_s > 0.2 \end{cases} \tag{S3}$$

Finally, the *WCS* was constructed as the geometric mean of the *TP* and *FC* sub-indices.

1.3 Wildlife Score (WLS)

The *WLS* is intended to capture biological condition, which is the similarity of the structure and function of biota to minimally disturbed condition (*sensu* Davies and Jackson 2006). More recently the EPA has been experimenting with a simpler metric: the O/E score, which is a ratio of the observed compared to expected numbers of taxa that would occur at a site if it were in reference condition (Hill et al. 2020). So high sites with high biological condition have an expected score of 1, where all the taxa expected at a minimally disturbed site are observed at the site being assessed. An advantage of the O/E score is that it is, apparently, easily conveyed to survey respondents, though it's meaning for the actual numbers of species will still vary by site based on natural variability. We plan to use other metrics to characterize similar attributes biological condition in our studies. However, WLS was not explicitly linked to underlying site-specific measurements or site-specific models for this part of our survey.

1.4 Water Quality Index (WQI)

As described in the main text, recall that McClelland's *WQI* is given by:[7]

[7] The notation here follows equation (2) in McClelland (1974, p. 16).

$$WQI = \prod_{i=1}^{n} q_i^{w_i} \quad \text{(S5)}$$

where q_i denotes the sub-index for water quality parameter i (as determined by the water quality “curves” elicited from the panel of experts); w_i denotes the weight assigned to water quality parameter i, where $\sum_{i=1}^{n} w_i = 1$; and n denotes the number of water quality parameters used in the index. The *WQI* is meant to capture the overall impact of water quality on water-based recreation, much like the traditional *WQI*’s that have evolved from McClelland’s (1974) work.

In our application and split-sample treatments, for the 2-index treatment that uses WQI and WLS, the WQI was constructed using the sub-indices FBS and WCS from the 3-index treatment. Thus the effects captured by our WQI include algal blooms, clarity, and water safety captured by the *WCS* and changes in fish biomass reflected by the *FBS*. As in S5, *WQI* is constructed as a weighted geometric average, but we use *WCS* and *FBS*, as the q_i with

$$WQI = FBS^{\omega} WCS^{1-\omega}, \quad \text{(S4)}$$

where we use $\omega = 0.33$ to reflect the relative importance of recreational fishing- versus nonfishing-based recreation in Michigan’s lower peninsula.

1.5 Ecological background on relationships between indices for changes in phosphorus

Changes in phosphorus concentrations, across the range of concentrations in most Michigan streams and lakes, would have opposite effects on FBS compared to WCS and WLS. With low phosphorus limiting productivity of most Michigan streams and lakes, an increase in phosphorus would decrease WCS by increasing algal growth (Vollenweider 1976; Quinlan et al. 2021), increasing risk of cyanobacterial blooms (Downing et al. 2001), reducing lake and river water clarity (Lambou et al. 1982), and increasing abundance of algae on stream bottoms and in lake littoral zones (Stevenson et al. 2006; Higgins et al. 2008).

In contrast to WCS, FBS would increase with increasing phosphorus because greater algae and the invertebrates produced by eating algae can increase fish growth. Fish biomass in most Michigan streams increases with phosphorus enrichment (Zorn and Nuhfer 2007; Esselman et al. 2015). Fish biomass in low nutrient lakes, like most lakes in Michigan, increase with phosphorus level (Downing et al. 1990; Jeppesen et al. 1994; Jacobson et al. 2017). The positive relationship between nutrients and fisheries production has strong causal foundation with support from relationships developed with comparisons of streams and lakes with different phosphorus concentrations (Downing et al. 1990; Jeppersen et al. 1994; Esselman et al. 2015), based on experiments with only phosphorus manipulated

(Deegan and Peterson 1992; LeBrasseur et al. 1979), and modeling from the Great Lakes (Kao et al. 2014, 2016). Fisheries in many regions of the Great Lakes are limited by phosphorus, in part because of water column phosphorus reductions (oligotrophication) by Dreissenid mussels and reduced phosphorus loading from agricultural and urban sources.

Even though game fish biomass would increase with phosphorus concentrations in most streams, lakes, and Great Lakes zones in Michigan, the species of game fish would change. Multiple changes in chemical and physical structure of water bodies cause shifts in fish species with phosphorus pollution, thereby decreasing WLS (Miltner and Rankin 1998; Jacobson et al. 2017). Increases in algal abundance can reduce dissolved oxygen concentrations, which has great effects on fish species present. Increases of algae on stream bottoms and decreases in water clarity alter fish nesting, feeding, and predator avoidance. Trout biomass is more sensitive to increasing phosphorus than bass and panfish biomass in Michigan streams (Esselman et al. 2015). Increasing algal abundance can also stimulate growth of salmonids in lakes, especially in larger lakes (Stockner and MacIsaac 1996; Kao et al. 2016); but smaller lakes are susceptible to reduced habitat suitability with oxygen concentrations in cold lake bottom water and decreasing cold bottom water habitat volume caused by lower lake thermoclines (Tanner 1960). So even highly sensitive salmonids can be positively affected by low levels of nutrient enrichment in nutrient poor habitats as long as changes in other elements of habitat suitability are not changed greatly. High levels of nutrients greatly decrease dissolved oxygen to levels in which only a few species survive, such as carp, but even those fish have value in some communities of the US and many countries around the world where they are an important food source. In Michigan, most anglers are using inland lakes and fishing for bass and panfish (Melstrom et al. 2015), so modest increases in phosphorus in Michigan lakes would likely increase biomass of both of those fisheries.

Increases in fish biomass and changes in fish species by phosphorus pollution would be considered increased deviations in the structure and function of biota from minimally disturbed condition, which is Davies and Jackson (2006) use to measure biological condition. Biological condition is our definition of Wildlife Score (WLS). In addition to fish, most kinds of biota would change with increasing phosphorus in Michigan streams and lakes (Soranno et al. 2008; Stevenson et al. 2008; Jacobson et al. 2017). Thus, increases in phosphorus increase FBS, but lower WCS and WLS. It should be noted that WLS was not varied in our contingent evaluation exercise comparing the WQI with WCS and FBS combined in one index or separated into two indices.

Tradeoffs in phosphorus management in water bodies can be important in policy development. Upgrades to a wastewater treatment plant reduced the production and biomass of trout in the AuSable River, one of Michigan's most revered trout fisheries (Merron 1982). Proposed phosphorus load reductions into the western basin of Lake Erie could reduce FBS in streams and lakes of those coastal Michigan watersheds and would likely reduce FBS of the western basin of Lake Erie based on phosphorus limitation of that fishery (Zhang et al. 2016) and responses of similarly shallow and enriched waters of Saginaw Bay, Lake Huron (Kao et al. 2014).

Returning lakes and streams to the phosphorus levels predicted for minimally disturbed conditions could reduce FBS, even though habitat suitability for non-game fish species and biomass characteristic of high biological condition would be restored. Lake restoration in Europe reduced fish biomass (Jeppesen et al. 2005). Thus, there is a fundamental tradeoff in key goals of the Clean Water Act. Restoring the nation's waters for physical, chemical, and biological integrity would likely reduce the ecosystems services of game fisheries, thus the protection and propagation of fish, and likely shellfish and many other forms of wildlife. Our research for more than a decade has been designed to reconcile this tradeoff by: determining the functional relationships between ecosystem services, pollutants, and human activities; integrating those relationships with economic and risk models of ecosystem service valuation; and informing a strategy of managing different waters for different uses based on providing optimal/high ecosystem services for both use and non-use benefits (Stevenson and Sabater 2010; Stevenson 2011; Esselman et al. 2015; Garnache et al. 2016; Melstrom et al. 2015; Lupi et al. 2020).

S2. Using WQI Index for changes in FBS and WCS

This section describes how to use the *WQI* to value changes in the underly indices. One of the primary reasons for dividing the *WQI* into component parts and presenting them separately to survey respondents is that policies may cause the individual indices to move in opposite directions. These opposing movements would be masked if only the composite *WQI* were presented. For example, one could envision improvements in the *WCS* for a waterbody that would at the same time lead to decrements to the *FBS*. To illustrate this issue, consider specifying a policy changing *WQI*, increasing it by ΔWQI. This change can be achieved by an infinite number of combinations of changes to *FBS* and *WCS*, but for any change in *WCS* (ΔWCS) can be solved for the corresponding change in *FBS* (ΔFBS) that hold *WQI* constant. Using the functional form for *WQI* Specifically, we have that

$$WQI_0 + \Delta_Q = (FBS_0 + \Delta_F)^{\frac{1}{3}}(WCS_0 + \Delta_C)^{\frac{2}{3}} \quad \text{(S6)}$$

where WQI_0, FBS_0, and WCS_0 denote the baseline levels for the respective indices, which can be rearranged to solve for ΔF (see also Figure 3 in main text). Rearranging equation (S6), one can show that:

$$\Delta_F = \frac{(WQI_0 + \Delta_Q)^3}{(WCS_0 + \Delta_C)^2} - FBS_0 . \quad \text{(S7)}$$

That is, given a target level for Δ_Q, one can specify an arbitrary level for Δ_C and there will be a value for Δ_F that will achieve the desired change to the aggregate index *WQI*.

S3. Econometric details

The econometric analysis of the survey referendum is couched in terms of a logit model of the vote for (y = 1) or against (y = 0) the proposed policy scenarios. In this setting, the outcome is assumed to be a function of a latent variable y^*, where

$$y^* = U^1 - U^0, \quad \text{(S8)}$$

with U^1 and U^0 denoting the individual's utility under proposed and baseline conditions, respectively. Further, let:

$$U^j = V^j - \epsilon^j = \tilde{\beta}_0^j + \beta_1 x_1^j + \cdots + \beta_K x_K^j + \epsilon^j \quad \text{(S9)}$$

where x^j_k denotes the kth explanatory variable under scenario k, ϵ^j follows a Type I extreme value distribution, and $V^j \equiv \tilde{\beta}_0^j + \beta_1 x_1^j + \cdots + \beta_K x_K^j$ denotes the representative conditional utility from choosing option j = 0,1. The individual is assumed to choose alternative 1 if $y^* \equiv U^1 - U^0 > 0$, where

$$\begin{aligned} y^* \equiv U^1 - U^0 = \Delta_U &= (\tilde{\beta}_0^1 - \tilde{\beta}_0^0) + \beta_1\Delta_1 + \cdots + \beta_K\Delta_K + (\epsilon^1 - \epsilon^0) \\ &= \beta_0 + \beta_1\Delta_1 + \cdots + \beta_K\Delta_K + \eta \\ &= \Delta_V + \eta, \end{aligned} \quad \text{(S10)}$$

where $\beta_0 \equiv \tilde{\beta}_0^1 - \tilde{\beta}_0^0$, $\eta \equiv \epsilon^1 - \epsilon^0$, $\Delta_V \equiv V^1 - V^0$ and $\Delta_k \equiv x^1{}_k - x^0{}_k$ denotes the change under the proposed policy scenario in the kth attribute affecting utility.[8] As usual, we observe the choice variable as $y_i = 1(y_i^* > 0)$, with

[8] The term Δ_V is what is typically referred to as the index function for the logit model.

$$Pr(y_i = 1) = \frac{exp(\Delta_V)}{1 + exp(\Delta_V)} \quad \text{(S11)}$$

Separate 2- and 3-Index Models

In the simplest model for the two-index case let

$$\Delta_V = \beta_{20} + \beta_{2P} P + \delta_{2Q}\Delta_Q + \delta_{2W}\Delta_W \quad \text{(S12)}$$

where P denotes the price of the policy scenario; and Δ_k denotes the change in water quality index k ($k = Q$ for the Water Quality Index, WQI, and = W for the Wildlife Score, WLS). The corresponding willingness to pay (WTP_2) for the policy scenario would be $WTP_2 = [\beta_{20} + \delta_{2Q}\Delta_Q + \delta_{2W}\Delta_W]/(-\beta_{2P})$, with a marginal WTP for a change in index k given by $MWTP_{2k} = -\delta_{2k}/\beta_{2P}$. For the parallel model in the three-index case let:

$$\Delta_V = \beta_{30} + \beta_{3P} P + \delta_{3C}\Delta_C + \delta_{3F}\Delta_F + \delta_{3W}\Delta_W \quad \text{(S13)}$$

where Δ_C denotes the change in the Water Contact Score (WCS) and Δ_F denotes the change in the Fish Biomass Score (FBS).

For robustness checks, the above model is repeated using a model where the various water quality indices are entered in a logarithmic form. This would, for example, replace (S12) with

$$\begin{aligned}\Delta_V &= \beta_{20} + \beta_{2P} P + \delta_{2Q} ln(\mathrm{I}_{Q1}/\mathrm{I}_{Q0}) + \delta_{2W} ln(\mathrm{I}_{W1}/\mathrm{I}_{W0}) \\ &= \beta_{20} + \beta_{2P} P + \delta_{2Q} ln(1 + \Delta_Q/\mathrm{I}_{Q0}) + \delta_{2W} ln(1 + \Delta_W/\mathrm{I}_{W0}), \quad \text{(S14)}\end{aligned}$$

where I_j^0 and I_j^1 denote the initial and proposed water quality indices for index j ($j = Q$ for the WQI and $j = W$ for the WLS). Similarly, for the three index cases we would have:

$$\begin{aligned}\Delta_V &= \beta_{30} + \beta_{3P} P + \delta_{3C} ln(\mathrm{I}_{C1}/\mathrm{I}_{C0}) + \delta_{3F} ln(\mathrm{I}_{F1}/\mathrm{I}_{F0}) + \delta_{3W} ln(\mathrm{I}_{W1}/\mathrm{I}_{W0}) \\ &= \beta_{30} + \beta_{3P} P + \delta_{3C} ln(1 + \Delta_C/\mathrm{I}_{C0}) + \delta_{3F} ln(1 + \Delta_F/\mathrm{I}_{F0}) + \delta_{3W} ln(1 + \Delta_W/\mathrm{I}_{W0}). \quad \text{(15)}\end{aligned}$$

S4. Additional survey details

4.1 Survey development, testing and evaluation

The survey development built heavily from the foundations laid by previous research including work by EPA and associated researchers, including the water quality ladder (Figure S1) developed by Vaughan (1986) and used by Carson and Mitchell (1993).

The survey conducted a series of steps to develop and test our survey instrument (Figure S2 summarizes the process). At the start, we discussed the work with colleagues in other

projects and especially at the EPA National Center for Environmental Economics. EPA economists shared with us where they were in the process of developing a nationwide water quality valuation study. In particular, at the time of this survey the EPA had done a series of focus groups across the country, and they provided materials to us and shared many insights. We leveraged and built from those efforts to move forward with an instrument for our test of indices in Michigan. Hill et al. (2020) documents some of that work and subsequent work they conducted after we developed and tested our survey.

After drafting an early instrument based on what we learned from EPA, we began a series of one-on-one cognitive interviews to evaluate the drafts, which were done iteratively with revisions between them (Kaplowitz et al. 2004) as described in the main text of the paper. Importantly, some of the cognitive interviews were done in the the early wave of a pre-test pilot study after a few changes were made in response to some early answers and comments in the pilot.

As Figure S2 indicates the pilot was conducted in two main parts, with the first occurring during summer of 2018. The first part of the pilot was rolled out in batches and always contained open-ended comment boxed for respondent feedback in key sections. We used the first pilot to evaluate models and response data as well as to get insights from the open-ended feedback comments during and after each batch. Based on these we made some minor changes but also tested those out with some cognitive interviews. The first part counted three different versions of the instruments which differed somewhat due to being updated based on knowledge gained in the pilot. The first pilot consisted of both treatments with 30 design versions across the respondents within each treatment, and it received 704 responses.

The second part of the pilot was solely to gather data on choices with which to estimate priors for the final survey experimental design (discussed below). The fourth round of the pilot survey only used the 3-index treatment to generate data for priors for the experimental design (since the 2-index treatment's *WQI* was not designed and was derived from the *WQI* function using the design levels for *WCS* and *FBS*). The first three rounds were used to test at larger scale the iterative versions of the instrument with 352, 91, and 261 responses consisting of both treatments and 30 design versions each across the first three pilot waves. The survey instrument used in the final wave was based on the 3-index treatment using an instrument virtually identical to the final survey with the addition of some worker authentication items in the beginning to ensure real MTurk workers that met survey conditions took the survey.

4.2 Experimental design of cost and water quality change levels

For the pilot and full surveys Ngene (ChoiceMetrics 2018) was used to generate an experimental design to improve the efficiency of the estimated preference parameters (though here we describe the design of the full survey). The design sought to minimize D-error subject to several conditions imposed on the design. By design, Ngene generates attribute pairings across scenarios that in a manner that avoid multicollinearity issues with the individual attributes shown to respondents across the scenarios and hence allows statistical identification of the separate effects of each attribute. Informally, a lower D-error indicates an experimental design with a combination of attribute levels that does a better job of extracting information from respondents to better identify the preference parameters for the attributes. Since our individual water quality scores changes were included a level for zero, it is possible for a naïve design to have a "no-change scenario" where each quality variable could be zero, so we applied constraints to exclude such implausible attribute combinations. We also used a Bayesian design with normally distributed priors for the preference parameters that we obtained based on results of the final pilot model to set priors and standard errors in the design.

Full scale implementation was begun in October 2018, with the survey being administered in six waves to the Qualtrics Panel. Each wave randomly assigned respondents to the treatments and there were no significant differences in these across waves. To potentially further enhance the efficiency of the design, we conducted the survey in 6 sequential waves. Using code prepared in advance we would quickly run our estimation logits after each wave, generate new Bayesian normal priors, and rerun the experimental design. We would then generate all the appropriate maps and tables for that design and update the Qualtrics instrument code. In all then, across the six waves and the two treatments with 30 scenarios each, which necessitated generating 736 unique maps embedded in survey across the treatments, scenarios, and waves.

4. 3 Survey flow summary

While the content and development of the survey was informed by EPA needs, the literature, and especially Johnston et al. (2017), the survey flow, content, and some wording mirrored the work on the Deepwater Horizon Oil Spill (Bishop et al. 2017). Figures S3 to S11 provide some of the key images for the flow up to the referendum question as described in the main, which we merely summarize here to avoid repetition with the main text.

In either the 2- or 3-index treatments, the information about each index was shown once, and then sequentially for each separate index in that treatment. On each survey webpage

showing an index, compact text and bullets were used to describe the image followed by a graphic of the color scale and descriptions of the scale (shown in Figure S3 to S6 for each index, even though indices were not introduced together as in the appendix). These indices preceded a map of the lower peninsula of Michigan with the HUC8s color-coded to reflect the index level for each, which were shown with text descriptions. There were also questions in each index section to encourage interaction (e.g., reading and using) with the information and promote understanding. See Figure S7 for an example. Note that in Figure S7 there is a link at the top for “What is a watershed?” to remind the respondents what the shaded areas of the map represented (see Figure S8).[9]

After the indices were presented, the idea of a plan to change water quality was introduced and followed with a table with summary data for the indices and maps showing the baseline (without plan) and changed (with plan) levels for each index in the treatment. Figure S9 shows this for one of the 30 designs in the 3-index treatment. The next page (Figure S10) introduced that there would be a vote on the plan and the plan comes with a cost. Thus, income was elicited here. Next, Figure S11 shows the reasons to vote for and against the plan, while Figure S12 shows the vote page. Note the summary table form just before is a key part of the vote question. Figure S14 shows results from an analysis of the open-ended reasons for the vote, which evidenced little misunderstanding/scenario rejection.

Figure S13 provides a visual of the voting shares of yes responses for each treatment, which generally slopes downward but appears to be non-monotonic at one cost level. However, since the experimental design seeks to be efficient, the different cost levels do not necessarily share the same quality changes. Hence the statistical models are needed to

[9] When the indices were described in the survey, the index WCS was for “water contact such as boating, wading and swimming” and specifically excluded fish and aquatic organisms, although it did mention water clarity and bacteria such as fecal coliform. The labels that went with the image used levels that mentioned boating, wading and swimming in a manner similar to the traditional water quality ladder but without gamefish. The recreational fishing index (FBS) was described in text and bullets that specified it was for “recreational fishing for game fish.” Several species examples were mentioned in both bullets and in the text describing levels that accompanied the state map of the index by HUC 8 watersheds (gamefish species mentioned include cold water species such as trout and salmon, cool water species such as walleye, and warm water species such as panfish and bass). Alternatively, the wildlife score (WLS) was described for other aquatic species stressing the number of native species and its measurement relative to its natural state before any changes due to human activity (e.g., pollution, development, etc). The more similar the aquatic wildlife measures are to their natural levels, the higher the wildlife score the water body receives. Finally, in the experimental design the movements in the three indices (WLS, WCS and FBS) were largely independent to enable their statistical effects so that across the 30 designs many people saw the changes in the indices that were very different and would not suggest correlation, including some with reductions in FBS.

judge "scope." Equality by treatment of the voting shares could not be rejected at any cost level.

4.4 Survey Summary Statistics

Table S1 details the sample distribution across the six survey rounds and the two- versus three-index formats. The distribution of surveys across the rounds are, as expected, not statistically different between two- and three-index formats. The same is largely true in terms of the characteristics of the two subsamples. Table S2 compares the demographic features of the two- and three-index respondents. The last column in the table includes p-values from Pearson's chi-squared tests regarding whether the two- and three-index samples are very similar in terms of the listed characteristics and are not statistically different from each other at a 10 percent level.

S5. Estimation results, alternative specifications, and valuation

Table S3 presents the estimation results for the 2- and 3-index treatments, all of which have a linear effect of water quality in the utility function. Columns 1 and 3 represent 2-index models with and without demographic variables, while columns 2 and 4 show the same for the 3-index model. Table S4 compares models for the 2- and 3-index treatment with both linear and nonlinear utility of water quality. To facilitate comparisons, it repeats estimation results for the linear models without demographics (columns 1 and 2) before showing them for the logarithmic quality models (columns 5 and 6). Table S5 reports the marginal valuations of the various quality index for the linear and logarithmic models with and without demographics (the models are in rows organized by index). The marginal values are all significantly different that 0 at 5% or better, and within an index are not significantly different across model specifications. Thus, for further analysis and Figures in the main text the linear model without demographics is used. Welfare results, test outcomes, and implications are qualitatively very similar across specifications.

The remainder of the supplement provides tables to support Figures 3 to 5 of the main text, which investigate the welfare differences for the 2- and 3-index models for evaluating changes in FBS and WCS which are not equal, yet achieve the same change in WQI(FBS, WCS) from equation (S4). Specifically, Table S6 presents the marginal values for a one unit change in FBS or WCS when the other index in WQI(·,·) is held constant. The top portion shows the results in the 2-index model in which the change in either FBS or WCS is mapped through WQI, whereas the bottom part repeats results for the 3-index model from the marginal values in Table S5. The results shown in Table S6 show clear differences that

result from marginal changes in either FBS or WCS when these are modeled directly as in the 3-index model or are funneled through WQI in the 2-index model. The results demonstrate that when a marginal change in only one underlying sub-index is mapped through WQI in the model with WQI alone, the value is uniformly smaller than the respective marginal value in the model with FBS and WCS modeled separately.

For completeness, Table S7 reports some combinations of FBS and WCS that yield the same change in WQI(FBS,WCS) from equation (S7), which are also plotted in Figure 3 of the main paper. Table S8 then reports the non-marginal WTP for some of the combinations of ΔWCS ΔFBS that yield these different levels of $\Delta WQI.$ There are sets of columns from the 2-index mode, the 3-index model and the difference between them. (The results in Table S8 form the bars in Figure 5 of the main text.) Looking across any row, we again see that the exact same underlying changes in FBS & WCS are valued lower, always, in the 2-index model than in the 3-index model.

Table S1: Distribution of survey completes by round and numbers of indices used

Round	Two-Index	Three-Index	Round Total
1	134	134	268
2	135	126	261
3	261	257	518
4	175	176	348
5	85	86	171
6	74	78	152
Total	861	857	1,718

Table S2: Treatment balance of demographics

Characteristic	Two-Index	Three-Index	Overall	p-value
Older Adult (Age>54) (%)	41.2	39.9	40.6	0.66
College Degree (%)	80.7	77.6	79.2	0.11
Gender (Female=1) (%)	74.6	73.4	74.0	0.58
Employed (%)	47.6	49.7	48.7	0.38
Fishing license (%)	18.9	21.0	20.0	0.28
Adults in household (#)	2.10	2.09	2.09	0.87
Income ($1000)	57.7	58.7	58.2	0.66

Table S3: Model results comparing 2- and 3-Index with and without demographics.

Variables	(1)<br>2-Index<br>Linear	(2)<br>3-Index<br>Linear	(3)<br>2-Index w/<br>Demographics	(4)<br>3-Index w/<br>Demographics
Cost	-0.0014***<br>(0.000)	-0.0017***<br>(0.000)	-0.0015***<br>(0.000)	-0.0017***<br>(0.000)
ΔWLS	0.0320***<br>(0.007)	0.0148**<br>(0.007)	0.0315***<br>(0.007)	0.0143**<br>(0.007)
Δ*WQI*	0.0374***<br>(0.012)		0.0400***<br>(0.012)	
Δ*WCS*		0.0537***<br>(0.009)		0.0545***<br>(0.009)
Δ*FBS*		0.0184***<br>(0.007)		0.0191***<br>(0.007)
Constant	0.4787***<br>(0.143)	0.3947***<br>(0.144)	-0.7505***<br>(0.287)	-0.0572<br>(0.278)
Income (thousands)			0.0065***<br>(0.002)	0.0042**<br>(0.002)
male			0.0136<br>(0.175)	-0.1706<br>(0.168)
college			0.4318**<br>(0.190)	0.1809<br>(0.178)
job			0.3577**<br>(0.152)	0.2247<br>(0.151)
Adults#			0.1594*<br>(0.091)	-0.004<br>(0.090)
Fish license			0.2475<br>(0.196)	0.1723<br>(0.187)
N	861	857	861	857
LogL	-545.024	-539.291	-523.3	-531.589
# of vars.	4	5	10	11
chi2	64.357	83.434	107.805	98.839
p-value	<0.000	<0.000	<0.000	<0.000
AIC	1098.049	1088.582	1066.601	1085.178
BIC	1117.081	1112.35	1114.182	1137.466

Note: ***, ** and * are 1%, 5% and 10* significance levels. Standard errors in parentheses.

Table S4: Model results comparing 2- and 3-Index with linear and ln quality.

Variables	(1)<br>2-Index<br>Linear(Q)	(2)<br>3-Index<br>Linear(Q)	(5)<br>2-Index<br>Ln(Q)	(6)<br>3-Index<br>Ln(Q)
Cost	-0.0014***<br>(0.000)	-0.0017***<br>(0.000)	-0.0014***<br>(0.000)	-0.0017***<br>(0.000)
ΔWLS	0.0320***<br>(0.007)	0.0148**<br>(0.007)	2.1879***<br>(0.477)	1.0438**<br>(0.478)
Δ*WQI*	0.0374***<br>(0.012)		2.7101***<br>(0.847)	
Δ*WCS*		0.0537***<br>(0.009)		4.0306***<br>(0.664)
Δ*FBS*		0.0184***<br>(0.007)		1.3240***<br>(0.511)
Constant	0.4787***<br>(0.143)	0.3947***<br>(0.144)	0.4627***<br>(0.145)	0.3918***<br>(0.144)
N	861	857	861	857
LogL	-545.024	-539.291	-545.05	-539.149
# of vars.	4	5	4	5
chi2	64.357	83.434	64.305	83.719
p-value	<0.000	<0.000	<0.000	<0.000
AIC	1098.049	1088.582	1098.101	1088.297
BIC	1117.081	1112.35	1117.133	1112.065

Note: ***, ** and * are 1%, 5% and 10* significance levels. Standard errors in parentheses.

Table S5: Model results comparing marginal values with and without demographics.†

Variable & Model f(Q)	Marginal value ($)	p-value	95% Confidence interval	
FBS				
Linear(Q)	11.00***	0.007	2.95	18.05
Linear(Q) w/ demographics	10.89***	0.006	3.13	18.64
Ln(Q)	16.63***	<0.000	7.64	25.61
Ln(Q) w/ demographics	12.03***	0.006	3.51	20.64
WCS				
Linear(Q)	32.09***	<0.000	22.29	41.88
Linear(Q) w/ demographics	30.90***	<0.000	21.54	40.26
Ln(Q)	47.48***	<0.000	37.19	57.79
Ln(Q) w/ demographics	35.51***	<0.000	24.83	46.19
WQI‡				
Linear(Q)	26.33***	<0.000	11.67	40.98
Linear(Q) w/ demographics	26.88***	<0.000	12.57	41.20
Ln(Q)	29.40***	<0.000	13.02	34.26
Ln(Q) w/ demographics	30.03***	<0.000	14.03	46.97
WLS 2-index				
Linear(Q)	22.47***	<0.000	12. 50	32.44
Linear(Q) w/ demographics	21.21***	<0.000	11.56	30.86
Ln(Q)	23.73***	<0.000	13.21	34.26
Ln(Q) w/ demographics	22.38***	<0.000	12.20	32.58
WLS 3-index				
Linear(Q)	8.86**	0.027	1.03	16.68
Linear(Q) w/ demographics	8.35**	0.030	0.80	15.90
Ln(Q)	17.06***	<0.000	10.14	23.96
Ln(Q) w/ demographics	9.01**	0.026	1.07	16.85

† Standard errors were computed using the delta method.

‡ Note that the reported marginal value *WQI* is for changes in *WQI*=1, which could be composed of many combinations of *WCS* and *FBS* (see Table S7).

Table S6: Model results comparing tests of constraints on marginal values implied by how *WQI* would work in practice when a sub-index changes.†

Variable & Model f(Q)	Marginal value ($)	p-value	95% Confidence interval	
2-index model: Marginal values of *FBS* & *WCS* via *WQI*(*WCS*,*FBS*)				
Δ*FBS*=1 and Δ*WCS*=0				
Linear(Q)	8.77	<0.000	3.89	13.65
Linear(Q) w/ demographics	8.95	<0.000	4.18	13.72
Ln(Q)	9.79	<0.000	4.34	15.24
Ln(Q) w/ demographics	9.99	<0.000	4.67	15.32
Δ*WCS*=1 and Δ*FBS*=0				
Linear(Q)	17.56	<0.000	7.79	27.33
Linear(Q) w/ demographics	17.96	<0.000	8.38	27.48
Ln(Q)	19.61	<0.000	8.69	30.53
Ln(Q) w/ demographics	20.03	<0.000	9.36	30.70
3-index model: Marginal values of *FBS* & *WCS* are given in rows 1-8 in Table S5				
Difference between 3-index values and 2-index values				
Δ*FBS*=1				
Linear(Q)	2.24	0.641	-7.18	11.66
Linear(Q) w/ demographics	1.93	0.068	-7.17	11.03
Ln(Q)	6.84	0.202	-3.67	17.35
Ln(Q) w/ demographics	2.03	0.692	-8.02	12.08
Δ*WCS*=1				
Linear(Q)	14.53	0.040	0.69	28.37
Linear(Q) w/ demographics	12.97	0.057	-0.39	26.32
Ln(Q)	27.88	<0.000	12.87	42.89
Ln(Q) w/ demographics	15.48	0.044	0.041	30.56

† Note the reported *WQI* is for changes in *WQI*(*WCS*,*FBS*) and when either *FBS* or *WCS* changes by 1. When Δ*WCS*=1 and Δ*FBS*=0, *WQI*(1,0)=0.667 and when Δ*WCS*=1 and Δ*FBS*=0, *WQI*(1,0)=0.333, the reflecting the respective exponent weights on the sub-indices in *WQI*. Standard errors were computed using the delta method.

Table S7: Level curves of ΔWCS and ΔFBS for various ΔWQI

ΔWQI	ΔWCS	ΔFBS
1	0	3.05
1	5	-6.33
1	10	-13.89
1	15	-20.08
1	20	-25.21
1	25	-29.51
1	30	-33.14
5	0	16.18
5	5	5.00
5	10	-4.02
5	15	-11.41
5	20	-17.53
5	25	-22.65
5	30	-26.99
10	0	34.85
10	5	21.10
10	10	10.00
10	15	0.92
10	20	-6.61
10	25	-12.92
10	30	-18.25
15	0	56.18†
15	5	39.49†
15	10	26.02
15	15	15.00
15	20	5.87
15	25	-1.79
15	30	-8.27
20	0	80.36†
20	5	60.33†
20	10	44.18†
20	15	30.96
20	20	20.00
20	25	10.82
20	30	3.05

† These values are not feasible since the max quality score would exceed 100.

Table S8: Differences in willingness to pay ($WTP) for combinations of Δ*FBS* and Δ*WCS* yielding various Δ*WQI* in the formula for *WQI*.†

			2-Index		3-Index		2-Index minus 3-Index	
Δ*WQI*	Δ*WCS*	Δ*FBS*	$\overline{\text{WTP}}_2$	s.e.	$\overline{\text{WTP}}_3$	s.e.	$\overline{\text{WTP}}_{2\text{-}3}$	s.e.
1	0	3.05	26.3	7.5	33.6	12.5	-7.2	14.6
1	5	-6.33	26.3	7.5	90.8	33.8	-64.4	34.6
1	10	-13.89	26.3	7.5	168.0	71.0	-141.7	71.4
1	15	-20.08	26.3	7.5	260.3	104.3	-234.0	104.6
1	20	-25.21	26.3	7.5	364.2	134.7	-337.9	134.9
1	25	-29.51	26.3	7.5	477.3	162.9	-451.0	163.1
1	30	-33.14	26.3	7.5	597.8	189.6	-571.5	189.7
5	0	16.18	131.6	37.4	178.1	66.5	-46.5	76.3
5	5	5.00	131.6	37.4	215.5	34.3	-83.8	50.7
5	10	-4.02	131.6	37.4	276.6	50.6	-145.0	62.9
5	15	-11.41	131.6	37.4	355.7	83.3	-224.1	91.3
5	20	-17.53	131.6	37.4	448.8	115.7	-317.1	121.6
5	25	-22.65	131.6	37.4	552.9	146.2	-421.2	150.9
5	30	-26.99	131.6	37.4	665.5	175.0	-533.9	179.0
10	0	34.85	263.3	74.8	383.6	143.3	-120.3	161.6
10	5	21.10	263.3	74.8	392.7	93.2	-129.4	119.5
10	10	10.00	263.3	74.8	430.9	68.6	-167.7	101.5
10	15	0.92	263.3	74.8	491.4	75.6	-228.2	106.3
10	20	-6.61	263.3	74.8	569.0	100.3	-305.7	125.1
10	25	-12.92	263.3	74.8	659.9	129.5	-396.7	149.6
10	30	-18.25	263.3	74.8	759.0	159.4	-495.7	176.1
15	0	56.18‡	394.9	112.1	618.4	231.0	-223.5	256.8
15	5	39.49‡	394.9	112.1	595.1	167.3	-200.2	201.4
15	10	26.02	394.9	112.1	607.3	123.6	-212.4	166.9
15	15	15.00	394.9	112.1	646.4	102.9	-251.5	152.2
15	20	5.87	394.9	112.1	706.3	105.8	-311.4	154.1
15	25	-1.79	394.9	112.1	782.4	124.3	-387.6	167.4
15	30	-8.27	394.9	112.1	871.6	149.6	-476.7	186.9

† Standard errors were computed using the delta method.

‡ These values are not technically feasible since the max quality score would exceed 100.

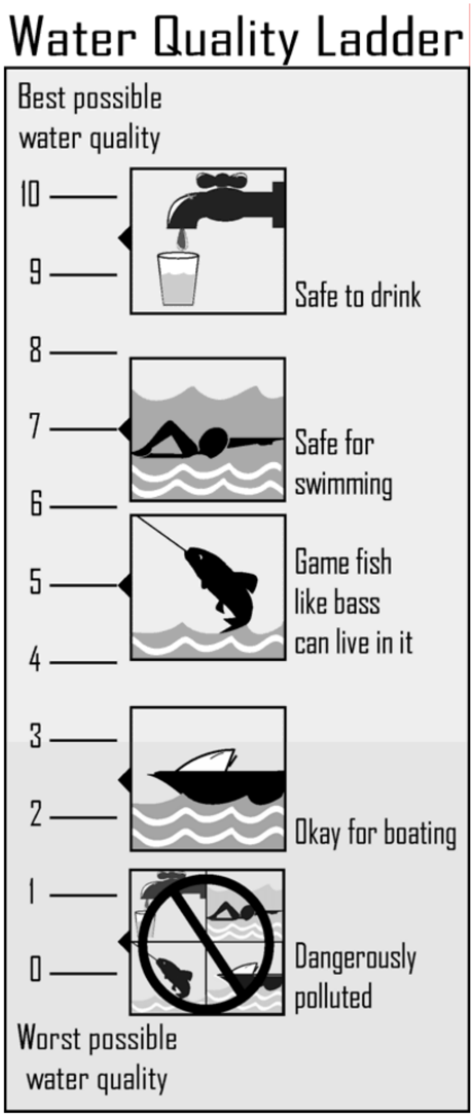

Figure S1: A classic Water Quality Ladder Wildlife (from Jeon et al. 2005)

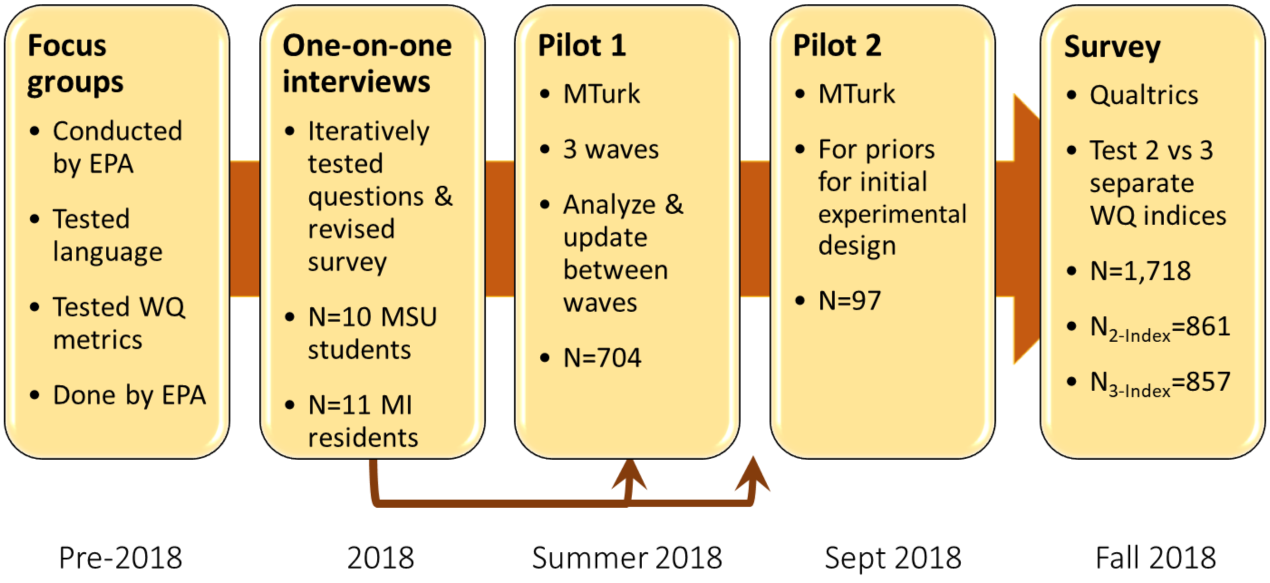

Figure S2: Survey development, testing, and implementation timeline

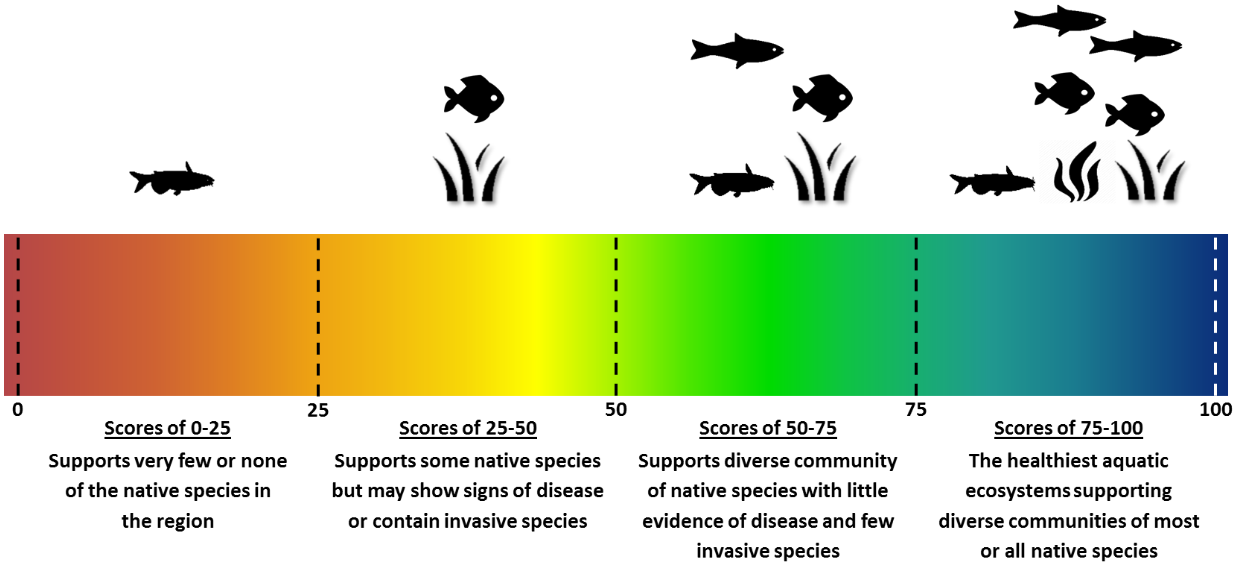

Figure S3: Wildlife score (WLS) used in both 2- and 3-index treatments

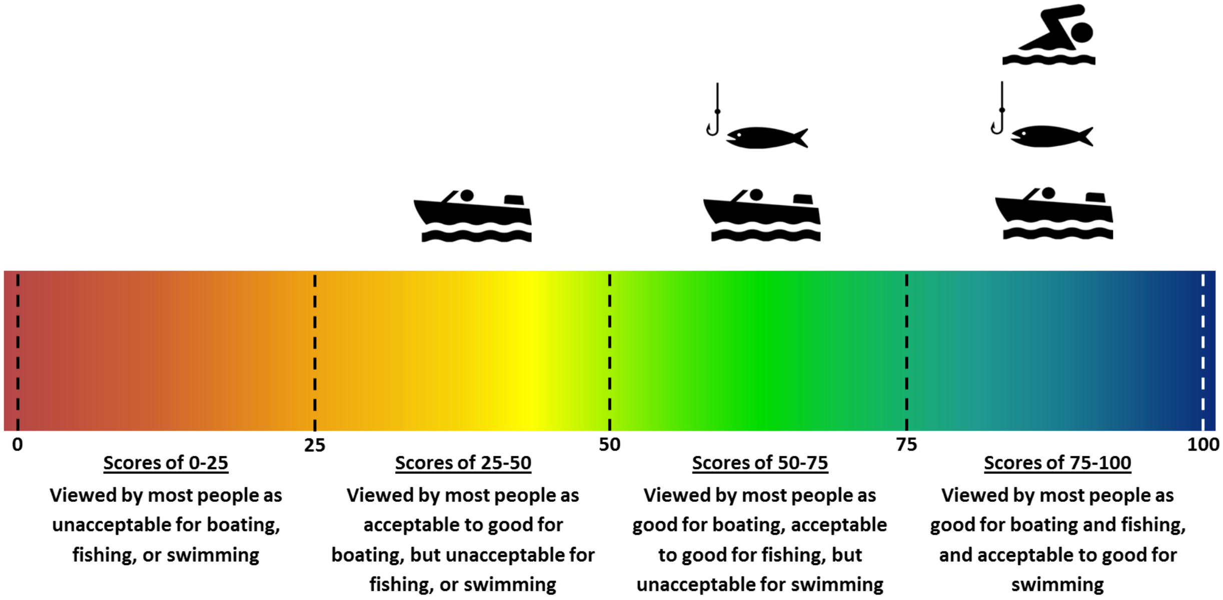

Figure S4: Water recreation score (WQI) used in 2-index treatment

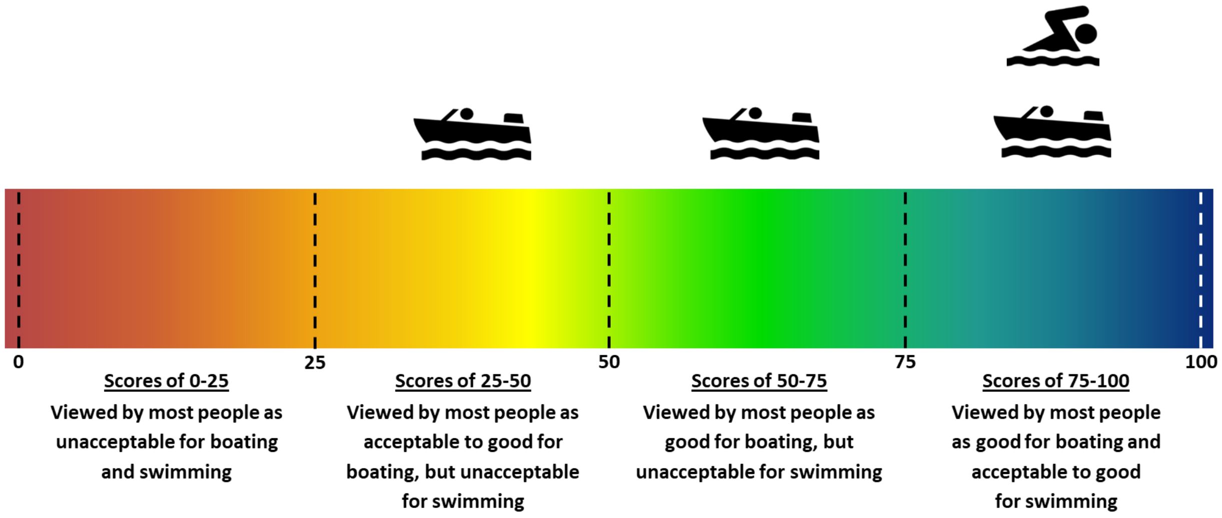

Figure S5: Water contact score (*WCS*) used in 3-index treatment

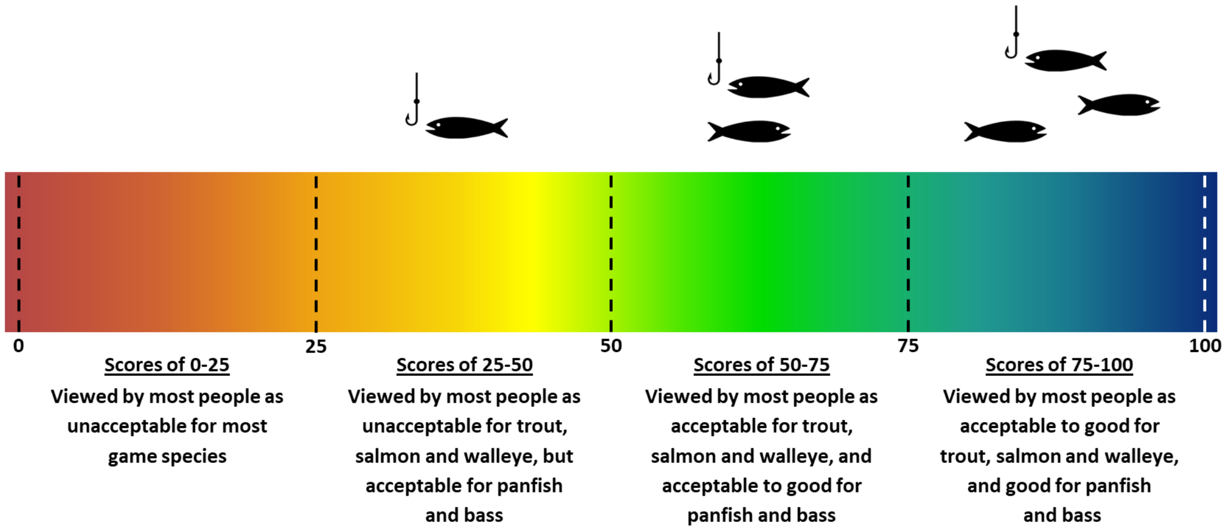

Figure S6: Fish biomass score (*FBS*) used in 3-index treatment

The map below shows the average Recreation Water Quality Scores for lakes, rivers, and streams in the Lower Peninsula of Michigan. The mapped areas represent average water quality scores for all lakes, streams and rivers within each watershed. What is a watershed?

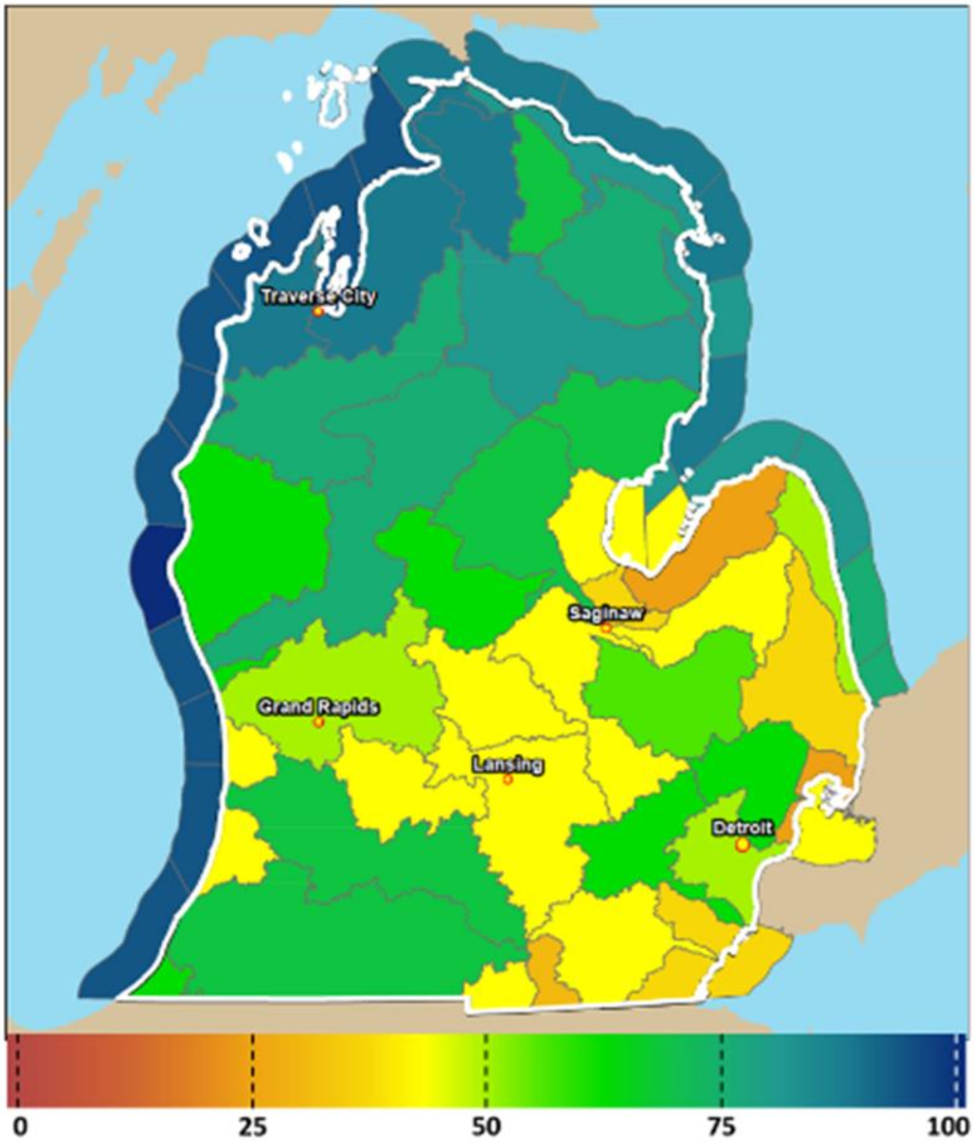

1. According to the map, what is the average Recreation Water Quality Score near your home?

Figure S7: Information example: Baseline *WQI* and an interaction question

Watersheds showing the land area that drains into a river or Great Lake. The maps will show quality for all lakes, rivers and streams within a watershed.

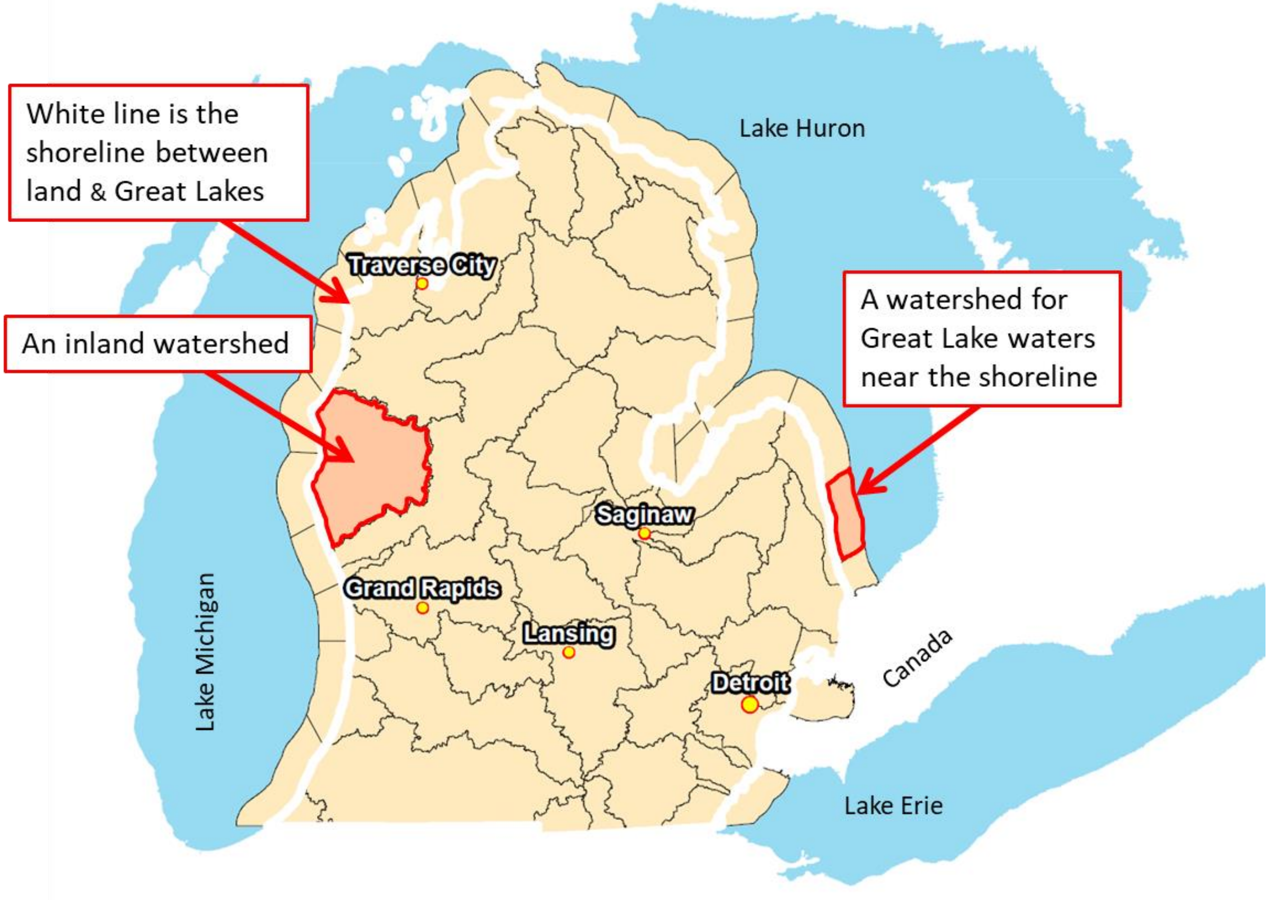

Figure S8: Information example: Watershed map to illustrate idea

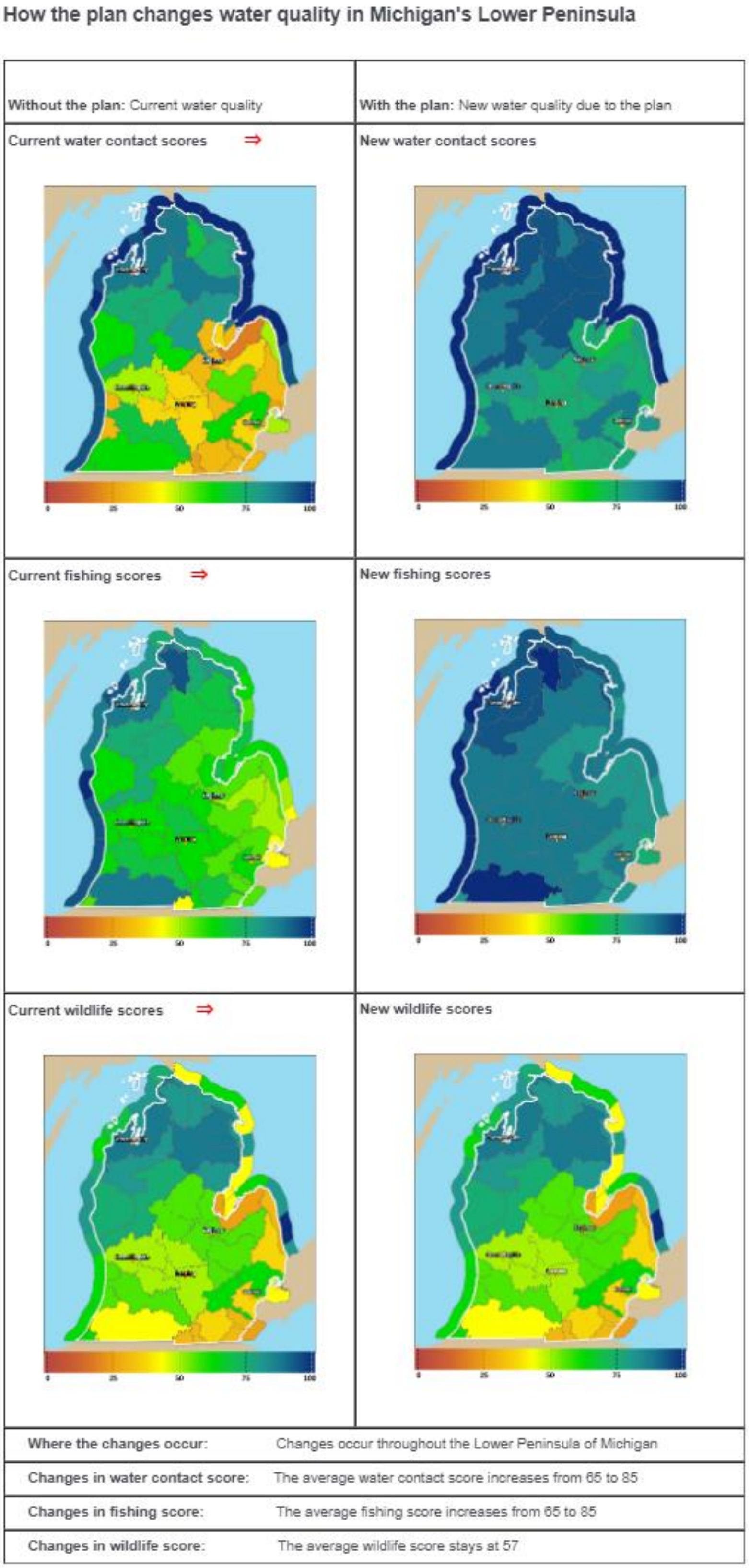

Figure S9: Information example: How the plan affects water quality

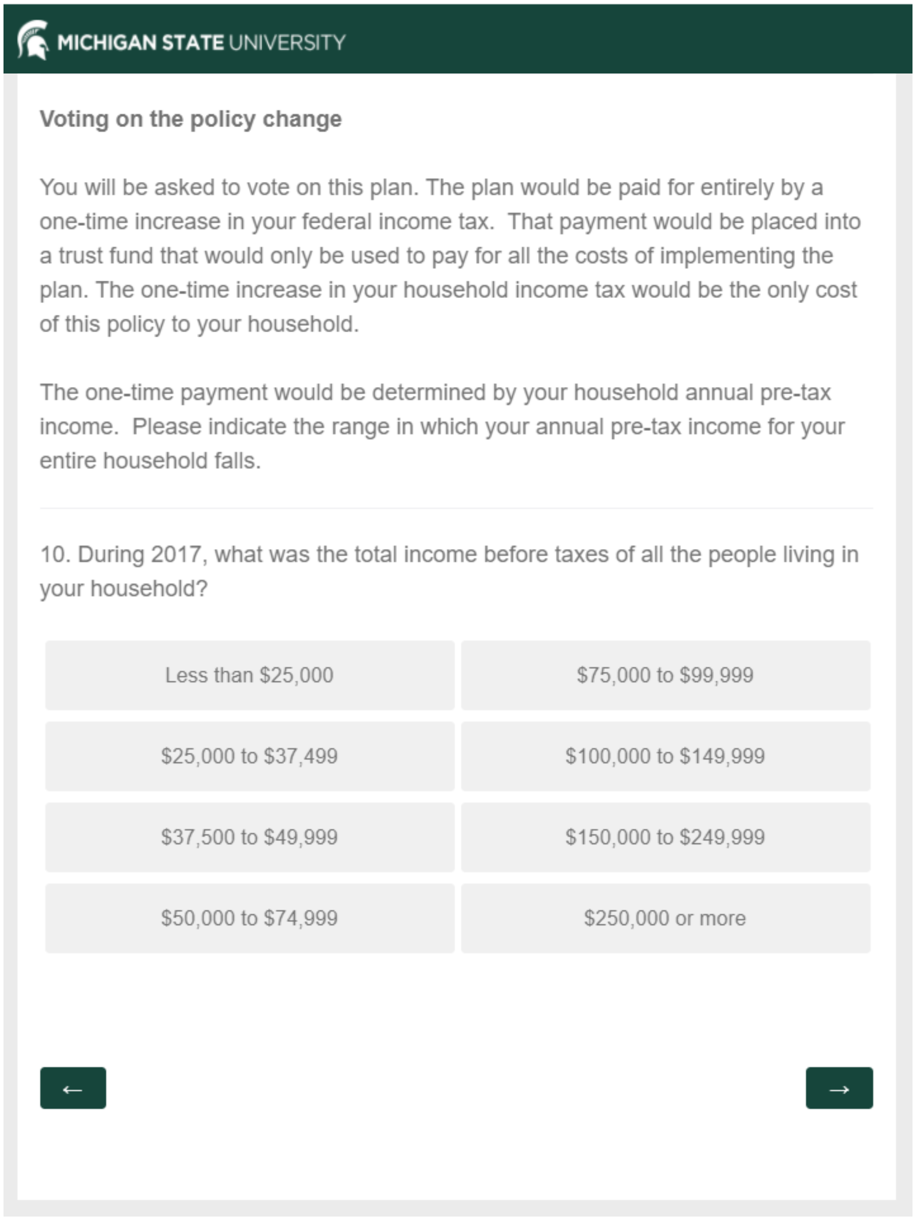

MICHIGAN STATE UNIVERSITY

Voting on the policy change

You will be asked to vote on this plan. The plan would be paid for entirely by a one-time increase in your federal income tax. That payment would be placed into a trust fund that would only be used to pay for all the costs of implementing the plan. The one-time increase in your household income tax would be the only cost of this policy to your household.

The one-time payment would be determined by your household annual pre-tax income. Please indicate the range in which your annual pre-tax income for your entire household falls.

10. During 2017, what was the total income before taxes of all the people living in your household?

Less than $25,000	$75,000 to $99,999
$25,000 to $37,499	$100,000 to $149,999
$37,500 to $49,999	$150,000 to $249,999
$50,000 to $74,999	$250,000 or more

← →

Figure S10: Plan payment information and income elicitation

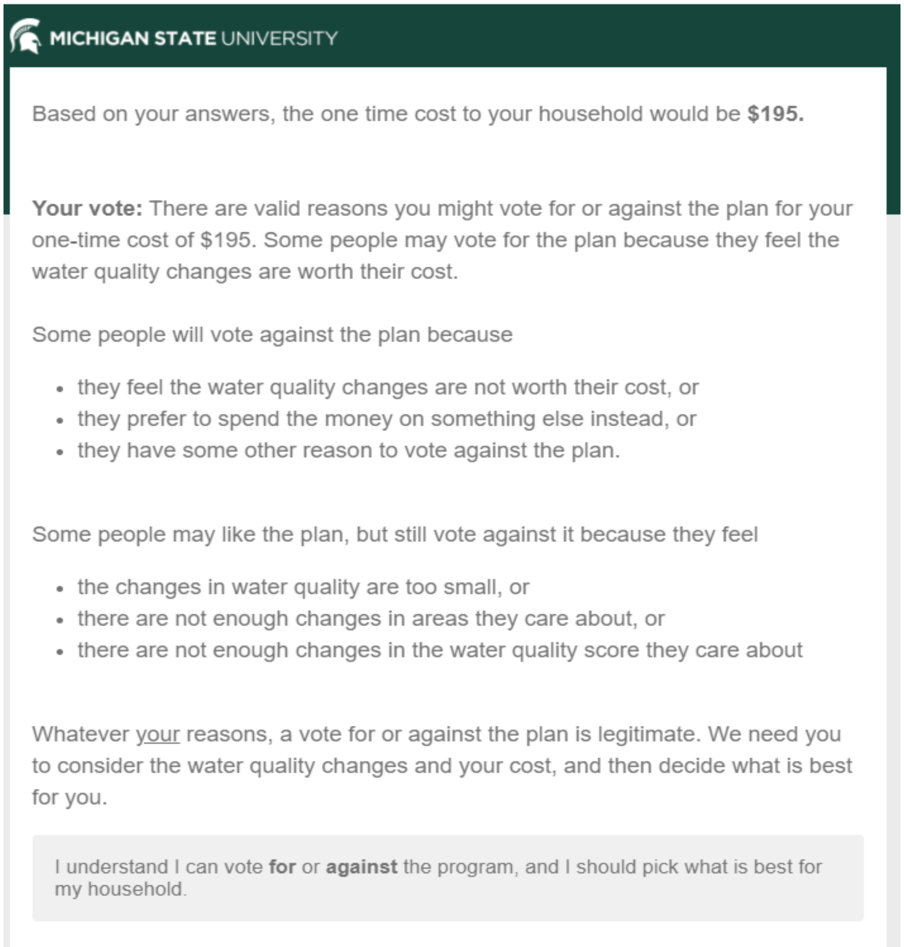

MICHIGAN STATE UNIVERSITY

Based on your answers, the one time cost to your household would be **$195.**

Your vote: There are valid reasons you might vote for or against the plan for your one-time cost of $195. Some people may vote for the plan because they feel the water quality changes are worth their cost.

Some people will vote against the plan because

- they feel the water quality changes are not worth their cost, or
- they prefer to spend the money on something else instead, or
- they have some other reason to vote against the plan.

Some people may like the plan, but still vote against it because they feel

- the changes in water quality are too small, or
- there are not enough changes in areas they care about, or
- there are not enough changes in the water quality score they care about

Whatever your reasons, a vote for or against the plan is legitimate. We need you to consider the water quality changes and your cost, and then decide what is best for you.

I understand I can vote **for** or **against** the program, and I should pick what is best for my household.

Figure S11: Information example: Reasons to vote for and against

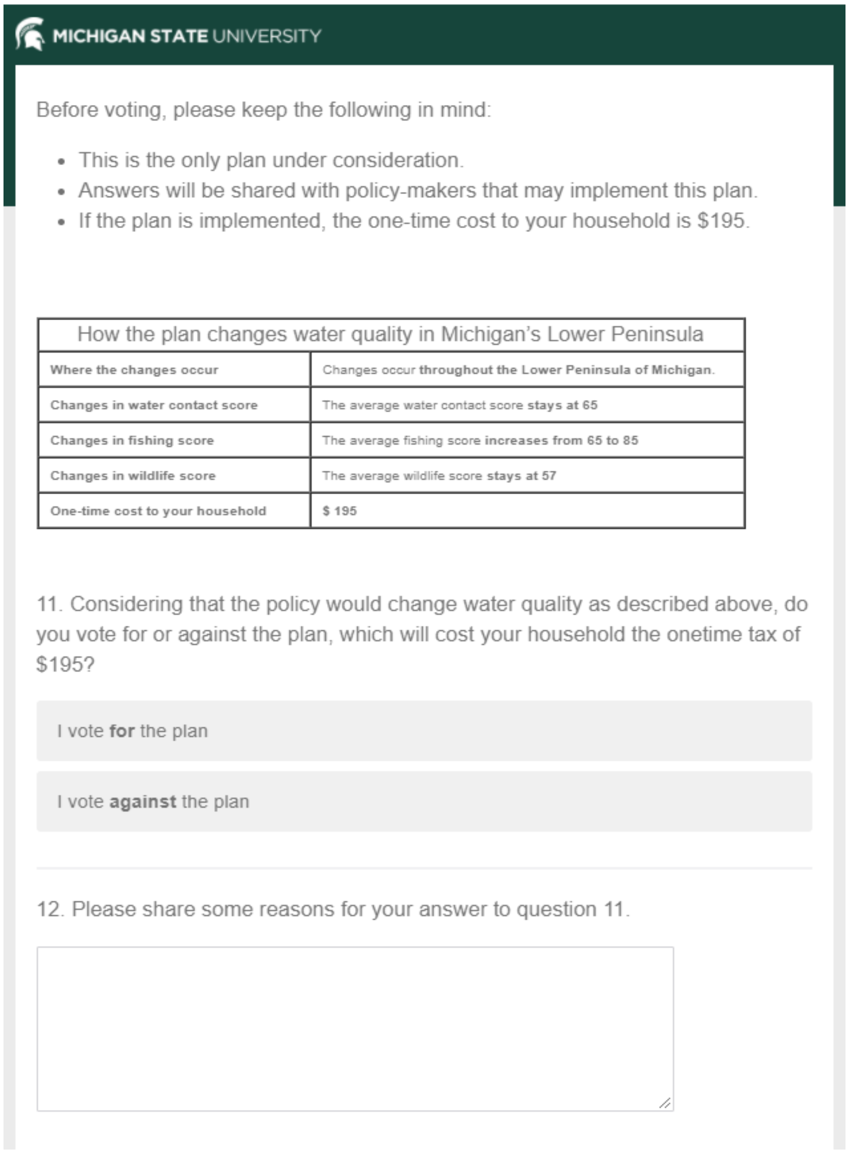

MICHIGAN STATE UNIVERSITY

Before voting, please keep the following in mind:

- This is the only plan under consideration.
- Answers will be shared with policy-makers that may implement this plan.
- If the plan is implemented, the one-time cost to your household is $195.

How the plan changes water quality in Michigan's Lower Peninsula	
**Where the changes occur**	Changes occur **throughout the Lower Peninsula of Michigan.**
**Changes in water contact score**	The average water contact score **stays at 65**
**Changes in fishing score**	The average fishing score **increases from 65 to 85**
**Changes in wildlife score**	The average wildlife score **stays at 57**
**One-time cost to your household**	**$ 195**

11. Considering that the policy would change water quality as described above, do you vote for or against the plan, which will cost your household the onetime tax of $195?

I vote **for** the plan

I vote **against** the plan

12. Please share some reasons for your answer to question 11.

Figure S12: Plan information reminder and voting question

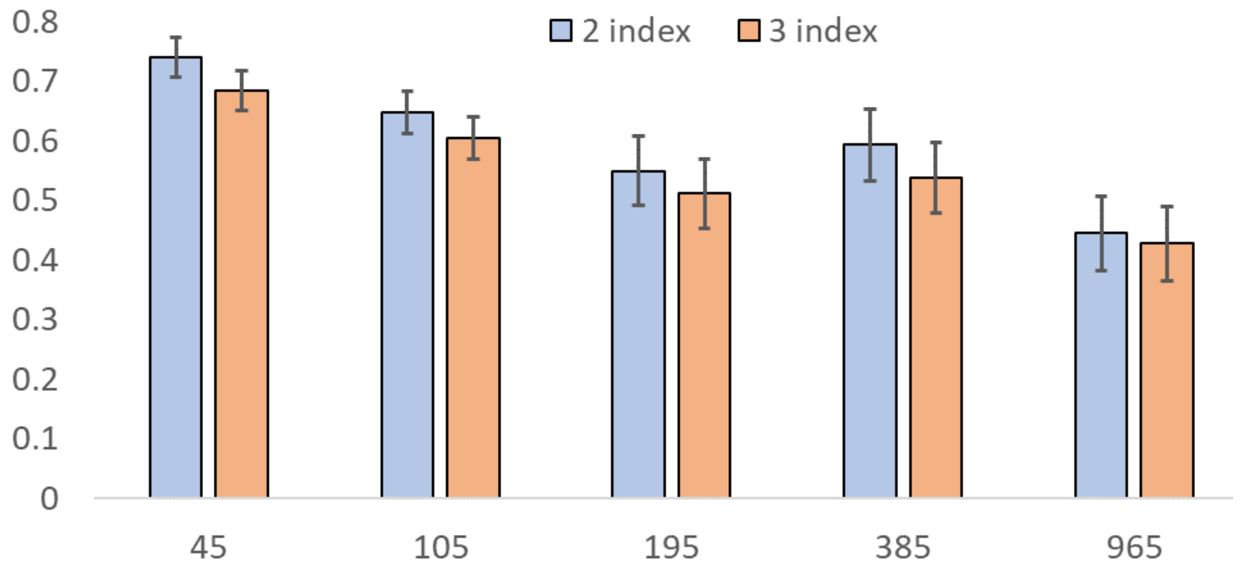

Figure S13: Votes for the plan tend to decline with higher payment. There is no significant difference in this response by 2 vs 3-index versions. Note that the percentage yes responses need not change monotonically with cost since the accompanying water quality changes differ at the different cost levels.

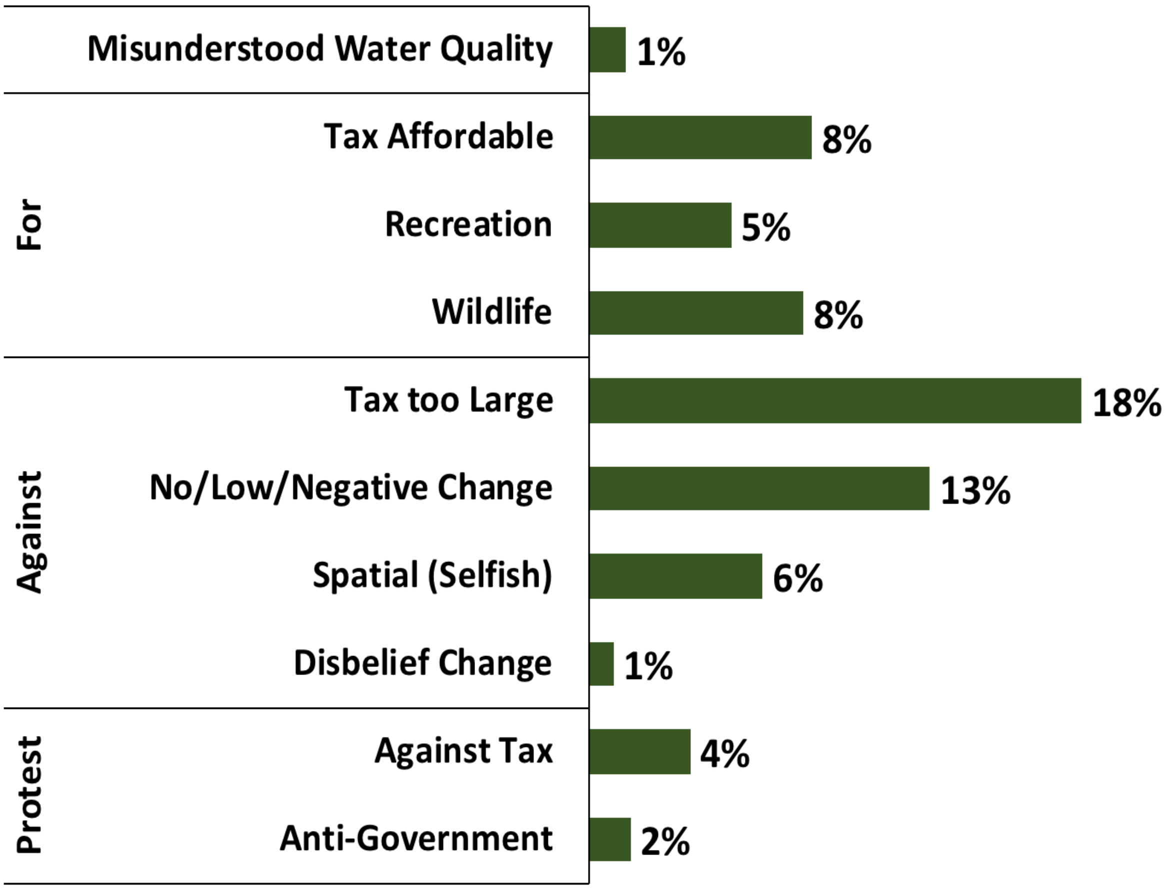

Figure S14: Summary of content analysis of open-ended comments for reasons for vote

Notes: The categories in Figure S14 are not mutually exclusive. Those respondents evidencing misunderstanding of the water quality scenario and disbelief of the scenario (i.e., scenario rejection) amounted to 1% each of the comments. Six percent could be considered protest votes, which we included in the analysis as valid votes against the change.

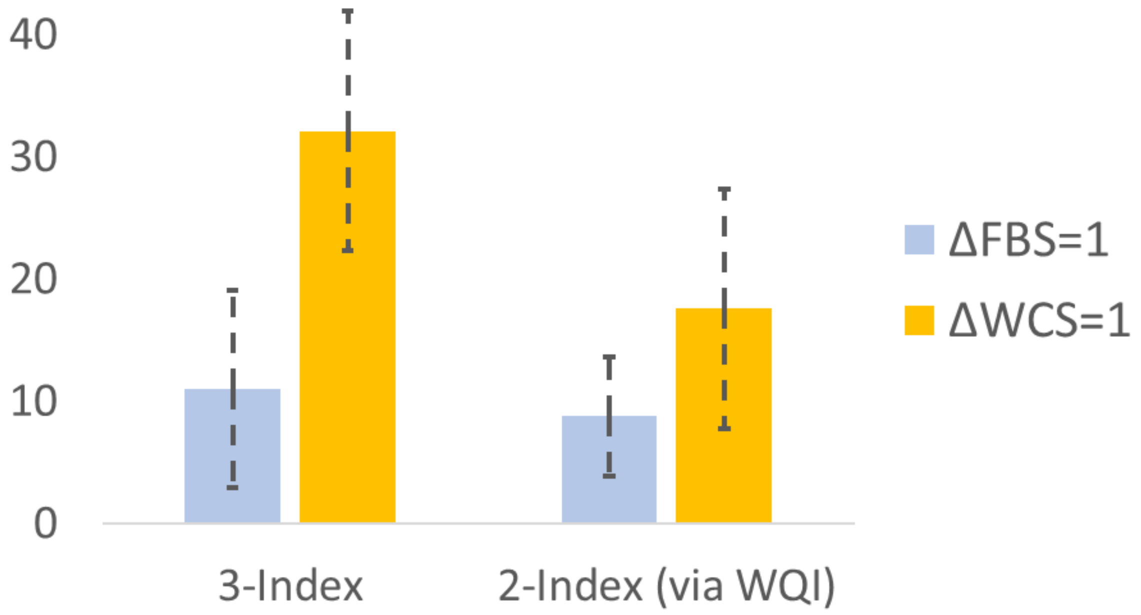

Figure S15: Marginal values of WCS and FBS in two versus three index models. For the two index model, a one unit change in WCS alone or FBS alone are each mapped through WQI. The shaded lines depict the 95% confidence intervals. The values for the change in WCS are significantly different across the models at the 90% level (Table S6).

S6. References

Allan J.D., P.B. McIntyre, S.D.P. Smith, B.S. Halpern, G.L. Boyer, A. Buchsbaum, G.A. Burton, L.M. Campbell, W.L. Chadderton, J.J.H. Ciborowski, P.J. Doran, T. Eder, D.M. Infante, L.B. Johnson, C.A. Joseph, A.L. Marino, A. Prusevich, J.G. Read, J.R. Rose, E.S. Rutherford, S.P. Sowa, and A.D. Steinman. 2013. Joint analysis of stressors and ecosystem services to enhance restoration effectiveness. Proceedings of the National Academy of Sciences 110:372–377

Bishop, R.C., K. J. Boyle, R.T. Carson, D. Chapman, W.M. Hanemann, B. Kanninen, ... & R. Paterson. 2017. Putting a value on injuries to natural assets: The BP oil spill. Science 356:253-254.

Carson, R. and R. Mitchell. 1993. The value of clean water: The public's willingness to pay for boatable, fishable, and swimmable quality water. Water Resources Research 29:2445-2454.

ChoiceMetrics. 2018. Ngene 1.2.1 User Manual and Reference Guide, Australia.

Cude, C.G. 2001. Oregon water quality index: A tool for evaluating water quality management effectiveness, Journal of the American Water Resources Association 37:125-137.

Davies, S.P. and S.K. Jackson. 2006. The biological condition gradient: A descriptive model for interpreting change in aquatic ecosystems. Ecological Applications 16(4): 1251-1266.

Deegan, L.A., and B.J. Peterson. 1992. Whole-river fertilization stimulates fish production in an Arctic tundra river. Canadian Journal of Fisheries and Aquatic Sciences 49:1890-1901.

Downing, J.A., and E. McCauley. 1992. The nitrogen - phosphorus relationship in lakes. Limnology and Oceanography 37:936-945.

Downing, J.A., S.B. Watson, and E. McCauley. 2001. Predicting Cyanobacteria dominance in lakes. Canadian Journal of Fisheries and Aquatic Sciences 58:1905-1908.

Dunnette, D.A. 1979. A geographically variable water quality index used in Oregon. Journal Water Pollution Control Federation 51:53-61.

Esselman, P., R.J. Stevenson, F. Lupi, C.R. Riseng, and M.J. Wiley. 2015. Landscape prediction and mapping of game fish biomass, an ecosystem service of Michigan rivers. North American Journal of Fish Management. 35:302-320.

Garnache, C., S.M. Swinton, S.A. Herriges, F. Lupi, and R. J. Stevenson 2016. Solving the phosphorus pollution puzzle: Synthesis and directions for future research. American Journal of Agricultural Economics. 98:1334-1359.

Higgins, S. N., S. Y. Malkin, E. Todd Howell, S. J. Guildford, L. Campbell, V. Hiriart-Baer, and R. E. Hecky. 2008. An ecological review of Cladophora glomerata (Chlorophyta) in the Laurentian Great Lakes. Journal of Phycology 44:839-854.

Hill, R., C. Moore, J. Doyle, S. Leibowitz, P. Ringold, and B. Rashleigh. 2020. Valuing Aquatic Ecosystem Health at a National Scale: Modeling Biological Indicators Across Space and Time. U.S. Environmental Protection Agency, National Center for Environmental Economics, Working Paper 20-04, November 2020.

Jacobson, P.C., G.J.A. Hansen, B.J. Bethke, and T.K. Cross. 2017. Disentangling the effects of a century of eutrophication and climate warming on freshwater lake fish assemblages. PLOS ONE 12:e0182667.

Jeon, Y., J.A. Herriges, C.L. Kling, and J.A. Downing. 2005. The role of water quality perceptions in modeling lake recreation demand. Iowa State University Department of Economics, Working Paper Series, Working Paper #05032. Ames: Iowa State University.

Jeppesen, E., J. P. Jensen, M. Søndergaard, and T. L. Lauridsen. 2005. Response of fish and plankton to nutrient loading reduction in eight shallow Danish lakes with special emphasis on seasonal dynamics. Freshwater Biology 50:1616-1627.

Jeppesen, E., M. Søndergaard, E. Kanstrup, B. Petersen, R. B. Eriksen, M. Hammershøj, E. Mortensen, J. P. Jensen, and A. Have. 1994. Does the impact of nutrients on the biological structure and function of brackish and freshwater lakes differ? Hydrobiologia 275:15-30.
Jeon, Y., J.A. Herriges, C.L. Kling, and J.A. Downing. 2005. The role of water quality perceptions in modeling lake recreation demand. Iowa State University Department of Economics, Working Paper Series, Working Paper #05032. Ames: Iowa State University.
Johnston, R.J., Elena. Besedin, R. Stapler. 2017. Enhanced geospatial validity for meta-analysis and environmental benefit transfer: An application to water quality improvements. Environmental and Resource Economics 68, 343–375.

Kao, Y.-C., S. Adlerstein, and E. Rutherford. 2014. The relative impacts of nutrient loads and invasive species on a Great Lakes food web: An Ecopath with Ecosim analysis. Journal of Great Lakes Research 40:35-52.

Kao, Y.-C., S.A. Adlerstein, and E.S. Rutherford. 2016. Assessment of top-down and bottom-up controls on the collapse of alewives (Alosa pseudoharengus) in Lake Huron. Ecosystems 19:803-831.

Kaplowitz, M., F. Lupi, and J. Hoehn, Multiple-methods for developing and evaluating a stated preference survey for valuing wetland ecosystems. Chpt. 24 In Questionnaire Development, Evaluation, and Testing Methods, (S. Presser, et al., eds). 503-524. Wiley, New Jersey. 2004

Lambou, V.W., S.C. Hern, W.D. Taylor, and L.R. Williams. 1982. Chlorophyll, phosphorus, secchi disk, and trophic state. Journal of the American Water Resources Association 18:807-813.

LeBrasseur, R.J., C.D. McAllister, and T.R. Parsons. 1979. Addition of nutrients to a lake leads to greatly increased catch of salmon. Environmental Conservation 6:187-190.

Lupi, F., B. Basso, C. Garnache, J. Herriges, D. Hyndman, and R. Stevenson. 2020. Linking agricultural nutrient pollution to the value of freshwater ecosystem services. Land Economics 96:493–509.

McClelland, N. 1974. Water Quality Index Application in the Kansas River Basin. EPA907/9-74-001, February.

Melstrom, R. and F. Lupi. 2013. Valuing recreational fishing in the great Lakes. North American Journal of Fisheries Management 33:1184-1193.

Melstrom, R., F. Lupi, P. Esselman and R.J. Stevenson. 2015. Valuing recreational fishing quality at rivers and streams. Water Resources Research 51:140–150.

Merron, G.S. 1982. Growth rate of brown trout (Salmo trutta) in areas of the Au Sable River, Michigan, before and after domestic sewage diversion. Michigan Department of Natural Resources. Fisheries Division. Fisheries Research Report No. 1900.

Miltner, R.J., and E.T. Rankin. 1998. Primary nutrients and the biotic integrity of rivers and streams. Freshwater Biology 40:145-58.

Quinlan, R., A. Filazzola, O. Mahdiyan, A. Shuvo, K. Blagrave, C. Ewins, L. Moslenko, D.K. Gray, C.M. O'Reilly, and S. Sharma. 2021. Relationships of total phosphorus and chlorophyll in lakes worldwide. Limnology and Oceanography 66:392-404.

Rier, S.T., and R.J. Stevenson. 2006. Response of periphytic algae to gradients in nitrogen and phosphorus in streamside mesocosms. Hydrobiologia 561:131-147.

Riseng, C.M., M.J. Wiley, and R.J. Stevenson. 2004. Hydrologic disturbance and nutrient effects on benthic community structure in midwestern US streams: a covariance structure analysis. Journal of the North American Benthological Society 23:309-326.

Soranno, P.A., K.S. Cheruvelil, R.J. Stevenson, S.L. Rollins, S.W. Holden, S. Heaton, and E. Torng. 2008. A framework for developing ecosystem-specific nutrient criteria: Integrating biological thresholds with predictive modeling. Limnology and Oceanography 53:773-787.

Stevenson, R.J. 2011. A revised framework for coupled human and natural systems, propagating thresholds, and managing environmental problems. Physics and Chemistry of the Earth 36:342-351.

Stevenson, R.J., and S. Sabater. 2010. Understanding effects of global change on river ecosystems: science to support policy in a changing world. Hydrobiologia 657:3-18.

Stevenson, R.J., S.T. Rier, C.M. Riseng, R.E. Schultz, and M.J. Wiley. 2006. Comparing effects of nutrients on algal biomass in streams in two regions with different disturbance regimes and with applications for developing nutrient criteria. Hydrobiologia 561:149-165.

Stockner, J.G., and E.A. MacIsaac. 1996. British Columbia Lake Enrichment Programme: Two decades of habitat enhancement for sockeye salmon. Regulated Rivers: Research & Management 12:547-561.

Tanner, H.A. 1960. Some Consequences of Adding Fertilizer to Five Michigan Trout Lakes. Transactions of the American Fisheries Society 89:198-205.

U.S. Environmental Protection Agency. 2009. *National Lakes Assessment: A Collaborative Survey of the Nation's Lakes*. EPA 841-R-09-001. Washington, DC: U.S. Environmental Protection Agency.

Vaughan, W.J., 1986 The water quality ladder. in The Use of Contingent Valuation Data for Benefit Cost Analysis in Water Pollution Control, ed. Mitchell, R.C., and R. T. Carson, Appendix B. CR-810224-02. Washington, DC: U.S. EPA Office of Policy and Evaluation.

Vollenweider, R.A. 1976. Advances in defining critical loading levels for phosphorus in lake eutrophication. Memorie dell' Istituto Italiano di Idrobiologia dott 33:53-83.

Walsh, P.J., and W. Wheeler. 2013. Water quality indices and benefit-cost analysis. Journal of Benefit-Cost Analysis 4:81–105.

Zhang, H., E.S. Rutherford, D.M. Mason, M.E. Wittmann, D.M. Lodge, X. Zhu, T.B. Johnson, and A. Tucker. 2019. Modeling potential impacts of three benthic invasive species on the Lake Erie food web. Biological Invasions 21:1697-1719.

Zorn, T.G., and A.J. Nuhfer. 2007. Influences on Brown Trout and Brook Trout Population Dynamics in a Michigan River. Transactions of the American Fisheries Society 136:691-705.
